# Comparative and phylogenetic analysis of the complete chloroplast genomes of six *Polygonatum* species (Asparagaceae)

**DOI:** 10.1038/s41598-023-34083-1

**Published:** 2023-05-04

**Authors:** Dongjuan Zhang, Jing Ren, Hui Jiang, Vincent Okelo Wanga, Xiang Dong, Guangwan Hu

**Affiliations:** 1 CAS Key Laboratory of Plant Germplasm Enhancement and Specialty Agriculture Wuhan Botanical Garden, Chinese Academy of Sciences, Wuhan, 430074 China; 2 Center of Conservation Biology, Core Botanical Gardens, Chinese Academy of Sciences, Wuhan, 430074 China; 3 Sino-Africa Joint Research Center, Chinese Academy of Sciences, Wuhan, 430074 China; 4 University of Chinese Academy of Sciences, Beijing, 100049 China; 5 College of Life Sciences, Hunan Normal University, Changsha, 410081 China

**Keywords:** Evolution, Genetics, Molecular biology, Plant sciences

## Abstract

*Polygonatum* Miller belongs to the tribe Polygonateae of Asparagaceae. The horizontal creeping fleshy roots of several species in this genus serve as traditional Chinese medicine. Previous studies have mainly reported the size and gene contents of the plastomes, with little information on the comparative analysis of the plastid genomes of this genus. Additionally, there are still some species whose chloroplast genome information has not been reported. In this study, the complete plastomes of six *Polygonatum* were sequenced and assembled, among them, the chloroplast genome of *P. campanulatum* was reported for the first time. Comparative and phylogenetic analyses were then conducted with the published plastomes of three related species. Results indicated that the whole plastome length of the *Polygonatum* species ranged from 154,564 bp (*P. multiflorum*) to 156,028 bp (*P. stenophyllum*) having a quadripartite structure of LSC and SSC separated by two IR regions. A total of 113 unique genes were detected in each of the species. Comparative analysis revealed that gene content and total GC content in these species were highly identical. No significant contraction or expansion was observed in the IR boundaries among all the species except *P. sibiricum*1, in which the *rps19* gene was pseudogenized owing to incomplete duplication. Abundant long dispersed repeats and SSRs were detected in each genome. There were five remarkably variable regions and 14 positively selected genes were identified among *Polygonatum* and *Heteropolygonatum.* Phylogenetic results based on chloroplast genome strongly supported the placement of *P. campanulatum* with alternate leaves in sect. *Verticillata*, a group characterized by whorled leaves. Moreover, *P. verticillatum* and *P. cyrtonema* were displayed as paraphyletic. This study revealed that the characters of plastomes in *Polygonatum* and *Heteropolygonatum* maintained a high degree of similarity. Five highly variable regions were found to be potential specific DNA barcodes in *Polygonatum*. Phylogenetic results suggested that leaf arrangement was not suitable as a basis for delimitation of subgeneric groups in *Polygonatum* and the definitions of *P. cyrtonema* and *P. verticillatum* require further study.

## Introduction

*Polygonatum* Miller belongs to the tribe Polygonateae Benth. & Hook. f. of the family Asparagaceae^1^. The species in this genus are perennial herbs with horizontal creeping fleshy roots and unbranched stems^2^. This genus comprises approximately 80 species in the world (https://wcsp.science.kew.org/), accessed 30 March 2022). According to Chen and Tamura^2^, 39 species have been recorded in China with 20 of them being endemic. *Polygonatum* is widely distributed in Northern Hemisphere, with the center of diversity in East Asia, especially in the Hengduan Mountains of southwest China and the eastern Himalayas^3,4^. This genus is valued significantly for its medicinal properties, with species such as *Polygonatum kingianum* and *P. sibiricum* being used as traditional Chinese medicine due to their properties of tonifying Qi, nourishing Yin, strengthening the spleen, moistening the lung and benefiting the kidney^5^.

Phylogenetic relationships reconstructed using ribosomal ITS and plastid DNA sequence suggested the monophyly of *Polygonatum* and its sister relationship to *Heteropolygonatum* M.N. Tamura & Ogisu^6–9^. In terms of infrageneric classification of this genus, it received considerable attention from researchers in history owing to the wide phenotypic variation within and among the species. Baker subdivided *Polygonatum* into three sections according to the leaf arrangement: the sect. *Alternifolia* with alternate leaves, sect. *Oppositifolia* with opposite leaves and sect. *Verticillata* with whorled leaves^10^. However, phyllotaxy types in this genus were considered to be unstable in subsequent studies^7^. On account of morphological traits like leaf arrangement, bract size and texture, length of the perianth tube, perianth shape, anther length and ovary shape, Tang et al. proposed eight series for *Polygonatum* distributed in China^11^. Based on karyological and micromorphological characters, Tamura sub-divided *Polygonatum* into the sect. *Polygonatum* and sect *Verticillata*^12^. Recently, Meng and Nie reconstructed the phylogenetic relationship among this genus using four chloroplast (cp) genes, *rbcL*, *trnK*, *trnC*-*petN* and *psbA*-*trnH,* and they proposed a new group on the basis of Tamura’s work, namely sect. *Sibirica*^7^. As a result, *Polygonaum* was divided into sect. *Polygonatum*, sect. *Verticillata* and sect. *Sibirica.* This infrageneric classification system was most widely accepted and was demonstrated by Floden’s research based on the complete cp genomes of *Polygonatum*^13,14^.

The chloroplast is a unique organelle found in green plants that is responsible for photosynthesis. It has a separate genome from the nuclear and the mitochondria genomes, and is mostly inherited matrilineally in angiosperms. Compared to the nuclear and the mitochondrial genomes, plastomes are small, less vulnerable to recombination, with low nucleotide substitution rates as well as generally more conserved in terms of gene structure and organization, and therefore can provide unique genetic information^15,16^. Among most higher plants, the cp genome possesses a typical tetrad structure comprising a small single-copy (SSC), a large single-copy (LSC), and two inverted repeats (IRs)^17^. Most cp genomes examined in plants have a constrained size varying from 120 to 160 kb, and this discrepancy is mainly related to expansion/contraction or even loss of IR^15,18,19^. Considerable genetic information is involved in the cp genome, which encodes about 120–130 genes^20^, which can be classified into three groups, genes involved in chloroplast gene expression, genes related to photosynthesis, and those with functions unclear^21^. The speed of molecular evolution between the coding and non-coding regions of chloroplast genomes differs noticeably, which is suitable for systematic studies at different levels^22^.

Benefiting from advances in next-generation sequencing technologies, cp genomes can be obtained more efficiently and economically. In the National Center for Biotechnology Information (NCBI) organelle genome database, there are about 40,000 cp genomes of plants currently published (accessed: 2023/1/21). Among angiosperms, plenty of cp genomes have been successfully employed to address the issues of phylogenetic relationships and species identification at different taxonomic levels^23–27^. There are about 156 complete cp genome sequences (ca. 40 species) of *Polygonatum* that have been reported in NCBI (accessed: 2022/11/07). However, previous studies have mainly been concerned with the size and gene contents of the plastid genome, with insufficient studies on the comparative genomic analysis^13,28^. Although, chloroplast gene fragments and complete cp genomes between species in *Polygonatum* have been adopted for phylogenetic analysis recently^7,13,28^, there are still some species whose complete chloroplast genome data have not been published, thus their phylogenetic placement of them is not well understood.

In this study, we reported the initial complete chloroplast genomes of *Polygonatum campanulatum*, together with the complete plastome sequences of *P. franchetii*, *P. cyrtonema*1, *P. filipes*1, *P. zanlanscianense*1 and *P. sibiricum*1, and then compared them with three related species i.e., *P. kingianum* (MW373517), *Heteropolygonatum alternicirrhosum* (MZ150832), *H. ginfushanicum* (MW363694). *P. campanulatum* is a critically endangered species discovered by professor Guangwan Hu in Yunnan Province in 2011. No molecular information about this species has been reported before, which can provide essential information for its conservation strategies and conducting restoration practices. In this present study, 9 species sequences were selected for plastid genome comparative analysis, including two species of *Heteropolygonatum* and seven species of *Polygonatum*, which covers the three subgroups of *Polygonatum* as well as the major branches. There are 11 plastomes of *Polygonatum* species that have not been verified by NCBI (Table S1). Manual checking of the 11 unverified plastomes found that the two IR regions had different lengths, and this discrepancy mainly occurred in the no-coding regions. Therefore, these unverified plastomes have only been used to reconstruct phylogenetic relationships and collect general information, and not for deep comparative analysis of the cp genome. A total of 56 published cp genome sequences (51 from *Polygonatum*; 4 from *Heteropolygonatum; Maianthemum henryi* was chosen as outgroup) obtained from the NCBI database were employed to reconstruct phylogenetic tree. The aims of this study were to (1) conducting a comprehensive analysis of the chloroplast genome among the six *Polygonatum* and its related species; (2) exploring hotspots regions of *Polygonatum* from the cp genomes; (3) inferring the phylogenetic relationships of *Polygonatum* species and determine the taxonomic status of *P. campanulatum*, *P. franchetii*, *P. cyrtonema, P. filipes, P. zanlanscianense* and *P. sibiricum* based on cp genome.

## Materials and methods

### Sample collection, total DNA extraction and sequencing

The six newly sequenced *Polygonatum* species (*Polygonatum campanulatum*, *P. filipes*, *P. franchetii*, *P. zanlanscianense*, *P. cyrtonema*, *P. sibiricum*) were collected by Guangwan Hu in China during the period of 2019 to 2021. Detailed field collection information of them is described in Table 1. The collected species were identified and verified by professor Guangwan Hu, from Wuhan Botanical Garden, Chinese Academy of Science. Voucher specimens were deposited at the Herbarium of Wuhan Botanical Garden, CAS (HIB) (China), with voucher specimen numbers listed in Table 1. Total genome DNA was extracted from the dry leaves preserved in silica gels, using a modified cetyltrimethylammonium bromide (CTAB) method, and then sequenced based on the Illumina HiSeq X Ten platform, 150 bp paired-end reads (PE150) at Novogene Co., Ltd. (Beijing, China).

**Table 1:** Specimen collection information of the six *Polygonatum* samples.

Species	Voucher specimen number	Date	Locality	Decimal latitude	Decimal longitude
*Polygonatum franchetii*	HGW-1223	2019-09-02	Gaowangjie National Nature Reserve, Guzhang County, Hunan Province, China	–	–
*P. zanlanscianense*	HGW-1357	2021-05-03	Malinyaozu Village, Xinning County, Hunan Province, China	26° 27ʹ 16ʹʹ N	110° 38ʹ 02ʹʹ E
*P. filipes*	HGW-1359	2021-05-03	Malinyaozu Village, Xinning County, Hunan Province, China	26° 27ʹ 12ʹʹ N	110° 38ʹ 52ʹʹ E
*P. sibiricum*	HGW-1379	2021-05-19	Donggang District, Rizhao City, Shandong Province, China	–	–
*P. campanulatum*	HGW-Z-2259	2019-07-27	Xima Township, Yingjiang County, Yunnan Province, China	24° 47ʹ 49ʹʹ N	97° 40ʹ 12ʹʹ E
*P. cyrtonema*	HGW-Z-2364	2020-08-19	Tiantangzhai Township, Jinzhai County, Anhui Province, China	31° 13ʹ 17ʹʹ N	115° 42ʹ 53ʹʹ E

### Assembly and annotation of chloroplast genome

Chloroplast genome assembling was done using Get Organelle v1.7.5^29^ with default parameters. Gene annotation was completed by PGA (Plastid Genome Annotator) software^30^ with *Amborella trichopoda* as a reference^31,32^. To ensure the reliability of the data used for subsequent analysis, all chloroplast genome download from NCBI was annotated over again by PGA. Manual checking and adjustment of the annotation results, including positions of initiation and termination codons and boundaries of IR repeat regions, were performed in Geneious v10.2.3^33^. Annotated chloroplast genome sequences of the six species were submitted to GenBank (Table S1) in NCBI. Further, the circular chloroplast genome map was drawn online by OGDRAW^34^.

### Comparative analysis of the whole chloroplast genome

Geneious v10.2.3^33^ was employed to analyze length and guanine-cytosine (GC) content of the whole chloroplast genome, LSC, SSC and IR regions, together with numbers of genes and genes categories. Multiple genome alignment analysis was performed in MAFFT program^35^. Comparative chloroplast genomes divergence was conducted and visualized by mVISTA^36^ with the annotation of *Polygonatum campanulatum* as a reference in Shuffle-LAGAN mode. To detect the contraction or expansion at the boundaries, the SC/IR boundary analysis of the chloroplast genomes was carried out by IRscope^37^. Mauve was adopted to perform the analyses of cp genome rearrangement based on default settings^38^, and one of the IR regions was removed uniformly in all sequences.

### Codon usage, and repeated sequences analysis

Relative synonymous codon usage (RSCU) value was detected using MEGA v7.0^39^. RSCU is defined as the ratio of the observed frequency of a codon to the expected frequency without preference. The values greater than 1.0 mean that the particular codons are used more frequently than expected, while the reverse indicates the opposite^40^.

Long dispersed repeats were identified using REPuter^41^ with a hamming distance equal to 3 bp, and repeat size no less than 30 bp. Simple sequence repeats (SSRs) were identified using MicroSatellite identification tool (MISA)^42^ with minimum parameters being set as 10, 5, 4, 3, 3, and 3 for mono-, di-, tri-, tetra-, penta-, and hexanucleotides SSR motifs, respectively.

### Nucleotide diversity analysis and selective pressure

DnaSP^43^ was adopted to analyze the nucleotide diversity (Pi) with the window length of 600 bp and the step size of 200 bp. Given that DnaSP v6 cannot recognize degenerate bases, like M, K, and Y, dashes were used to take the place of these letters. Further, the figure was generated in Excel and optimized in Adobe Illustrator.

To identify the positive selection loci of coding sequences (CDS) in the cp genome, the dN/dS values were calculated by employing EasyCodeML v1.12^44^. Each single-copy CDS were extracted from the complete chloroplast genome using Geneious v10.2.3^33^, after aligning under the codon model, they were finally combined into one matrix. The input tree was an ML tree reconstructed by IQ-TREE^45^. Four site models (i.e., M0 vs. M3, M1a vs. M2a, M7 vs. M8, and M8a vs. M8) along with a likelihood ratio test (LRT) were used to perform the analyses. Naive Empirical Bayes (NEB) and Bayes Empirical Bayes (BEB)^46^ analyses were conducted under the M8 model to identify positive selection loci and the selected genes.

### Phylogenetic analysis

The phylogenetic analysis was performed based on the complete chloroplast genomes of 57 *Polygonatum* sequences and 4 *Heteropolygonatum* taxa. *Maianthemum henryi* was set as an outgroup. The chloroplast genomes of all species were obtained from GenBank (Table S1), except for *Polygonatum campanulatum, P. filipes*1, *P. franchetii*, *P. zanlanscianense*1, *P. cyrtonema*1, and *P. sibiricum*1. The total matrix was aligned using MAFFT^35^. ModelFinder^47^ was adopted to select the best-fit model according to the Bayesian information criterion (BIC). Maximum likelihood (ML) phylogenetic tree was reconstructed using IQ-TREE^45^ under the GTR+I+G model for 5000 ultrafast bootstraps^48^. BI (Bayesian inference) analysis was conducted using MrBayes v3.2.6^49^ based on GTR+F+I+G4 model. Two independent Markov Chain Monte Carlo (MCMC) run for 1,000,000 generations, trees were sampled every 100 generations, and the initial 25% of sampled data were discarded as burn-in. The two output trees were visualized and improved by Figtree v1.4 (http://github.com/rambaut/figtree/).

### Ethical approval and consent to participate

The authors have complied with the relevant institutional, national and international guidelines in collecting biological materials for the study. The study contributes to facilitating future studies in population genetics and species identification.

## Results

### Chloroplast genome structure and characteristics analyses

**Figure 1:**
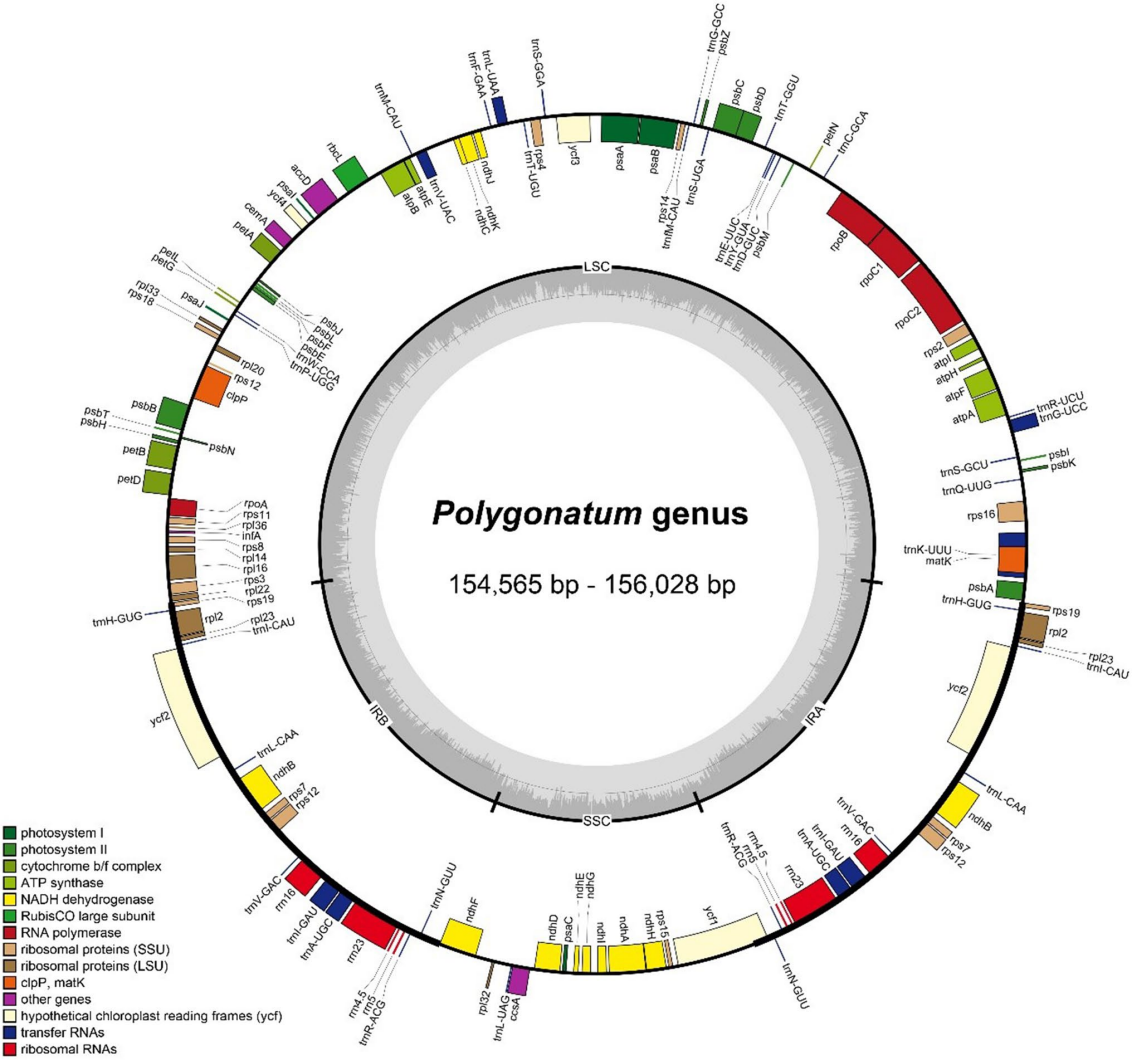
Gene map of the chloroplast genome among the *Polygonatum* species. Genes inside and outside the circle transcribed in counter-clockwise and clockwise respectively. The dark gray and light gray areas inside the inner circle indicate GC content and AT content respectively. LSC (Large single-copy), SSC (Small single-copy) and the inverted repeats (IRa, IRb) were denoted inner the circle.

The complete chloroplast genomes of the six newly sequenced species in *Polygonatum* displayed closed circular and common tetrad structures (Fig. 1). The length of the 57 cp genomes in *Polygonatum* ranged from 154,564 bp (*P. multiflorum*) to 156,028 bp (*P. stenophyllum*), while the length of the 4 cp genomes in *Heteropolygonatum* ranged from 155,436 (*H. pendulum*) to 155,944 (*H. alternicirrhosum*) (Table1). Each plastome included a large single-copy (LSC), a small single-copy (SSC) and a pair of inverted repeats (IRa and IRb) that separated the LSC and SSC regions (Fig. 1). The LSC regions of the *Polygonatum* species ranged from 83,486 bp (*P. odoratum*) to 94,843 bp (*P. sibiricum*3), while SSC regions varied from 18,210 bp (*P. cyrtonema*6) to 18,570 bp (*P. kingianum*2). The sizes of the IR regions ranged from 42,290 bp (*P. sibiricum*3) to 52,830 bp (*P. cirrhifolium, P. curvistylum*, *P. hookeri*, *P. prattii*, *P. verticillatu*3, *P. zanlanscianense*1, *P. zanlanscianense*2, *P. zanlanscianense*3) (Table 2). The total Guanine-Cytosine (GC) content of the plastomes ranged from 37.6 to 37.8%. Further, GC content exhibited an unbalanced distribution among the regions both in the cp genomes of *Polygonatum* and *Heteropolygonatum*. The SSC regions had presented the lowest GC content of 31.4% to 31.7%, followed by LSC regions (35.6–36.1%), whereas the IRs had the highest GC content ranging from 42.9 to 43% (Table 2).

**Table 2:** General information and comparison of chloroplast genomes of the 57 cp genomes of *Polygonatum* and 4 cp genomes of *Heteropolygonatum*.

Species	Genome length (bp)				GC content (%)					Gene number		
	Total	LSC	SSC	IR	Total	LSC	SSC	IR	Total	PCG	tRNA	rRNA
*H. alternicirrhosum*	155,944	84,968	18,520	52,456	131 (113)	85 (79)	38 (30)	8 (4)	37.6	35.6	31.5	43.0
*H. ginfushanicum*	155,508	84,552	18,528	52,428	132 (113)	86 (79)	38 (30)	8 (4)	37.6	35.6	31.4	43.0
*H. ogisui*	155,665	84,784	18,533	52,348	132 (113)	86 (79)	38 (30)	8 (4)	37.6	35.6	31.4	43.0
*H. pendulum*	155,436	84,609	18,365	52,462*	132 (113)	86 (79)	38 (30)	8 (4)	37.7	35.7	31.7	43.0
*P. acuminatifolium*	155,354	84,271	18,455	52,628	132 (113)	86 (79)	38 (30)	8 (4)	37.7	35.8	31.6	43.0
*P. annamense*	155,277	84,340	18,422	52,515*	132 (113)	86 (79)	38 (30)	8 (4)	37.7	35.7	31.6	43.0
*P. biflorum*	155,470	84,291	18,469	52,710*	132 (113)	86 (79)	38 (30)	8 (4)	37.7	35.7	31.6	43.0
***P. campanulatum***	155,487	84,458	18,373	52,656	132 (113)	86 (79)	38 (30)	8 (4)	37.6	35.7	31.7	42.9
*P. cirrhifolium*	155,944	84,568	18,546	52,830	132 (113)	86 (79)	38 (30)	8 (4)	37.6	35.7	31.5	42.9
*P. curvistylum*	155,939	84,563	18,546	52,830	132 (113)	86 (79)	38 (30)	8 (4)	37.6	35.7	31.5	42.9
***P. cyrtonema1***	155,509	84,448	18,303	52,758	132 (113)	86 (79)	38 (30)	8 (4)	37.7	35.7	31.7	43.0
*P. cyrtonema2*	155,512	84,462	18,292	52,758	132 (113)	86 (79)	38 (30)	8 (4)	37.7	35.7	31.7	42.9
*P. cyrtonema3*	155,164	84,023	18,495	52,646	132 (113)	86 (79)	38 (30)	8 (4)	37.7	35.7	31.6	43.0
*P. cyrtonema4*	155,614	84,452	18,420	52,742	132 (113)	86 (79)	38 (30)	8 (4)	37.7	35.7	31.7	43.0
*P. cyrtonema5*	155,044	83,896	18,498	52,650	132 (113)	86 (79)	38 (30)	8 (4)	37.7	35.7	31.6	43.0
*P. cyrtonema6*	155,205	84,457	18,210	52,538	132 (113)	86 (79)	38 (30)	8 (4)	37.7	35.7	31.7	43.0
***P. filipes1***	155,361	84,307	18,454	52,600	132 (113)	86 (79)	38 (30)	8 (4)	37.7	35.7	31.6	43.0
*P. filipes2*	155,334	84,280	18,454	52,600	132 (113)	86 (79)	38 (30)	8 (4)	37.7	35.7	31.6	43.0
*P. filipes3*	155,317	84,262	18,455	52,600	132 (113)	86 (79)	38 (30)	8 (4)	37.7	35.7	31.6	43.0
*P. filipes4*	155,337	84,262	18,455	52,620	132 (113)	86 (79)	38 (30)	8 (4)	37.7	35.7	31.6	43.0
***P. franchetii***	155,962	84,722	18,566	52,674	132 (113)	86 (79)	38 (30)	8 (4)	37.7	35.7	31.5	43.0
*P. govanianum*	155,089	84,212	18,228	52,649*	132 (113)	86 (79)	38 (30)	8 (4)	37.7	35.7	31.6	43.0
*P. hirtum*	155,490	84,385	18,419	52,686	132 (113)	86 (79)	38 (30)	8 (4)	37.7	35.7	31.6	42.9
*P. hookeri*	155,976	84,600	18,546	52,830	132 (113)	86 (79)	38 (30)	8 (4)	37.7	35.7	31.5	42.9
*P. hunanense1*	155,618	84,448	18,426	52,744	132 (113)	86 (79)	38 (30)	8 (4)	37.7	35.7	31.5	42.9
*P. hunanense2*	155,609	84,438	18,427	52,744	132 (113)	86 (79)	38 (30)	8 (4)	37.7	35.7	31.6	42.9
*P. hunanense3*	155,608	84,437	18,427	52,744	132 (113)	86 (79)	38 (30)	8 (4)	37.7	35.7	31.5	42.9
*P. hunanense4*	155,618	84,448	18,426	52,744	132 (113)	86 (79)	38 (30)	8 (4)	37.7	35.7	31.5	42.9
*P. inflatum*	154,898	84,270	18,454	52,174	132 (113)	86 (79)	38 (30)	8 (4)	37.7	35.7	31.6	43.0
*P. involucratum1*	155,370	84,280	18,450	52,640	132 (113)	86 (79)	38 (30)	8 (4)	37.7	35.7	31.6	43.0
*P. involucratum2*	155,372	84,282	18,450	52,640	132 (113)	86 (79)	38 (30)	8 (4)	37.7	35.7	31.6	43.0
*P. kingianum1*	155,826	84,627	18,547	52,652	132 (113)	86 (79)	38 (30)	8 (4)	37.7	35.7	31.6	43.0
*P. kingianum2*	155,824	84,632	18,570	52,622	132 (113)	86 (79)	38 (30)	8 (4)	37.7	35.7	31.5	43.0
*P. kingianum3*	155,824	84,626	18,546	52,652	132 (113)	86 (79)	38 (30)	8 (4)	37.7	35.7	31.6	43.0
*P. macropodum*	154,610	83,554	18,464	52,592	132 (113)	86 (79)	38 (30)	8 (4)	37.7	35.8	31.6	43.0
*P. mengtzense*	155,498	84,498	18,469	52,531*	132 (113)	86 (79)	38 (30)	8 (4)	37.7	35.7	31.5	43.0
*P. multiflorum*	154,564	83,525	18,457	52,582	132 (113)	86 (79)	38 (30)	8 (4)	37.7	35.8	31.5	43.0
*P. nodosum*	155,205	84,143	18,422	52,640	132 (113)	86 (79)	38 (30)	8 (4)	37.7	35.7	31.6	43.0
*P. odoratum*	154,569	83,486	18,459	52,624	132 (113)	86 (79)	38 (30)	8 (4)	37.8	35.8	31.6	43.0
*P. oppositifolium*	155,760	84,471	18,544	52,745*	132 (113)	86 (79)	38 (30)	8 (4)	37.6	35.7	31.4	42.9
*P. orientale*	155,386	84,225	18,456	52,705*	132 (113)	86 (79)	38 (30)	8 (4)	37.7	35.7	31.5	43.0
*P. prattii*	155,915	84,538	18,547	52,830	132 (113)	86 (79)	38 (30)	8 (4)	37.7	35.7	31.5	42.9
*P. punctatum*	155,657	84,542	18,423	52,692	132 (113)	86 (79)	38 (30)	8 (4)	37.7	35.7	31.5	43.0
***P. sibiricum1***	155,514	84,537	18,415	52,562	132 (113)	86 (79)	38 (30)	8 (4)	37.7	35.7	31.7	43.0
*P. sibiricum2*	155,514	84,542	18,416	52,556	131 (113)	85 (79)	38 (30)	8 (4)	37.7	35.7	31.7	43.0
*P. sibiricum3*	155,549	94,843	18,416	42,290	132 (113)	86 (79)	38 (30)	8 (4)	37.7	36.1	31.7	43.0
*P. sibiricum4*	155,512	84,533	18,417	52,562	131 (113)	85 (79)	38 (30)	8 (4)	37.7	35.7	31.7	43.0
*P. stenophyllum*	156,028	84,677	18,561	52,790	132 (113)	86 (79)	38 (30)	8 (4)	37.7	35.7	31.6	43.0
*P. stewartianum*	155,867	84,540	18,559	52,768*	131 (113)	85 (79)	38 (30)	8 (4)	37.7	35.7	31.5	42.9
*P. tessellatum1*	155,688	84,488	18,564	52,636	132 (113)	86 (79)	38 (30)	8 (4)	37.6	35.7	31.5	42.9
*P. tessellatum2*	155,688	84,488	18,564	52,636	132 (113)	86 (79)	38 (30)	8 (4)	37.6	35.7	31.5	42.9
*P. tessellatum3*	155,724	84,485	18,497	52,742*	132 (113)	86 (79)	38 (30)	8 (4)	37.6	35.7	31.4	42.9
*P. urceolatum*	155,504	84,492	18,435	52,577*	132 (113)	86 (79)	38 (30)	8 (4)	37.7	35.7	31.5	43.0
*P. verticillatum1*	155,589	84,242	18,523	52,824	132 (113)	86 (79)	38 (30)	8 (4)	37.7	35.7	31.6	42.9
*P. verticillatum2*	155,856	84,545	18,523	52,788	132 (113)	86 (79)	38 (30)	8 (4)	37.7	35.7	31.5	42.9
*P. verticillatum3*	155,505	84,207	18,468	52,830	132 (113)	86 (79)	38 (30)	8 (4)	37.7	35.7	31.5	42.9
*P. yunnanense*	155,363	84,229	18,427	52,707*	132 (113)	86 (79)	38 (30)	8 (4)	37.7	35.7	31.6	43.0
***P. zanlanscianense1***	155,787	84,418	18,539	52,830	132 (113)	86 (79)	38 (30)	8 (4)	37.7	35.7	31.6	42.9
*P. zanlanscianense2*	155,911	84,650	18,431	52,830	132 (113)	86 (79)	38 (30)	8 (4)	37.6	35.6	31.5	42.9
*P. zanlanscianense3*	155,911	84,650	18,431	52,830	132 (113)	86 (79)	38 (30)	8 (4)	37.6	35.6	31.5	42.9

The length of the two IR regions is different in this sequence. Newly sequenced species in this study are highlighted in bold.

A total of 131–132 genes (113 unique genes) were detected in the complete cp genomes of the 57 *Polygonatum* in the same order. One *rps19* gene was detected pseudogenized in *P. stewartianum* and *P. sibiricum*1 and *P. sibiricum*2. And, both *ycf1* genes were detected pseudogenized in *H. ogisui*. The whole genomes included 87 protein-coding genes (PCGs), 38 transfer RNA (tRNA) genes and 8 ribosomal RNA (rRNA) genes (Table 2). Moreover, a total of 19 genes, comprising 7 PCGs (*rps19*, *rpl2*, *rpl23*, *ycf2*, *ndhB*, *rps7*, *rps12*), 8 tRNA genes (*trnN-GUU*, *trnR-ACG*, *trnA-UGC*, *trnI-GAU*, *trnV-GAC*, *trnL-CAA*, *trnI-CAU*, *trnH-GUG*) and 4 rRNA genes (*rrn5*, *rrn4.5*, *rrn23*, *rrn16*) were duplicated in the pair of inverted repeats. In addition, a total of 18 genes (*trnA-UGC*, *trnG-UCC*, *trnI-GAU*, *trnK-UUU*, *trnL-UAA*, *trnV-UAC*, *rps12*, *rps16*, *rpl2*, *rpl16*, *rpoC1*, *petB*, *petD*, *atpF*, *ndhA*, *ndhB*, *clpP*, *ycf3*) and six tRNA contained at least one intron in the complete cp genome, in which *clpP* and *ycf3* included two introns. Particularly, *rps12* gene was a trans-spliced gene with the 5' exon situated in the LSC region and the two copies of 3' exon and intron sitting in the IRs. The longest intron was identified in *trnK-UUU* with the length of 2,568–2,586 bp and the *matK* gene was placed inside the intron (Table S2). All of the functional genes can be divided into three categories, i.e., self-replication genes, photosynthesis genes, and other genes (Table 3).

**Table 3:** The annotated genes in the chloroplast genomes of *Polygonatum*.

Category	Gene group	Gene name
Self-replication	Ribosomal RNA	*rrn4.5*^c^*, rrn5*^c^*, rrn16*^c^*, rrn23*^c^
	Transfer RNA	*trnA-UGC*^a,c^*, trnC-GCA, trnD-GUC, trnE-UUC, trnF-GAA, trnG-GCC, trnG-UCC*^a^*, trnH-GUG*^c^*, trnI-CAU*^c^*, trnI-GAU*^a,c^*, trnK-UUU*^a^*, trnL-CAA*^c^*, trnL-UAA*^a^*, trnL-UAG, trnM-CAU, trnfM-CAU, trnN-GUU*^c^*, trnP-UGG, trnQ-UUG, trnR-UCU, trnR-ACG*^c^*, trnS-UGA, trnS-GCU, trnS-GGA, trnT-GGU, trnT-UGU, trnV-UAC*^a^*, trnV-GAC*^c^*, trnW-CCA, trnY-GUA*
	Small subunit of ribosome	*rps2, rps3, rps4, rps7*^c^*, rps8, rps11, rps12*^a,c^*, rps14, rps15, rps16*^a^*, rps18, rps19*^c^
	Large subunit of ribosome	*rpl2*^a,c^*, rpl14, rpl16*^a^*, rpl20, rpl22, rpl23*^c^*, rpl32*^a^*, rpl33, rpl36*
	RNA polymerase subunits	*rpoA, rpoB, rpoC1*^a^*, rpoC2*
Photosynthesis	Photosystem I	*psaA, psaB, psaC, psaI, psaJ*
	Photosystem II	*psbA, psbB, psbC, psbD, psbE, psbF, psbH, psbI, psbJ, psbK, psbL, psbM, psbN, psbT, psbZ*
	Subunits of cytochrome	*petA, petB*^a^*, petD*^a^*, petG, petL, petN*
	ATP synthase	*atpA, atpB, atpE, atpF*^a^*, atpH, atpI*
	NADH-dehydrogenase	*ndhA*^a^*, ndhB*^a,c^*, ndhC, ndhD, ndhE, ndhF, ndhG, ndhH, ndhI, ndhJ, ndhK*
Other genes	Rubisco large subunit	*rbcL*
	Translational initiation	*infA*
	Maturase	*matK*
	Envelope membrane protein	*cemA*
	Acetyl-CoA-carboxylase	*accD*
	Proteolysis	*clpP*^b^
	c-type cytochrome synthesis gene	*ccsA*
	Conserved open reading frames	*ycf1, ycf2*^c^*, ycf3*^b^*, ycf4*

^a^Genes with one intron.

^b^Genes with two introns.

^c^Two genes copied in IR regions.

### Relative synonymous codon usage analysis

**Figure 2:**
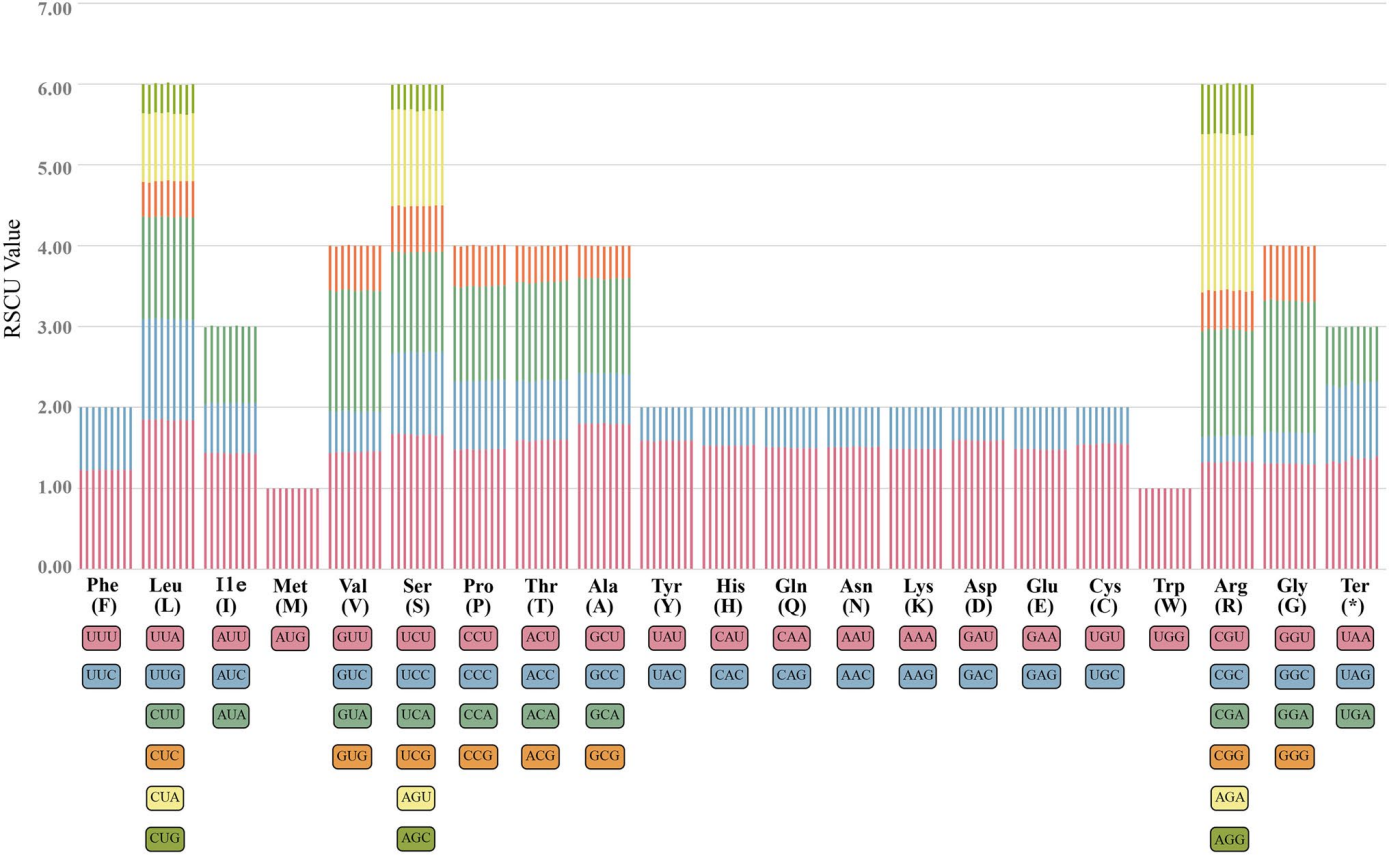
Relative synonymous codon usage (RSCU) value of 20 amino acids and stop codons of seven *Polygonatum* and two *Heteropolygonatum* species based on protein-coding sequences in chloroplast genomes. The colors of the bar correspond to the colors of codons. Each amino acid corresponds to nine histograms, and y-axis represents the RSCU value. The order of each six columns from left to right is *P. campanulatum*, *P. filipes*1, *P. franchetii*, *P. zanlanscianense*1, *P. cyrtonema*1, *P. sibiricum*1, *P. kingianum* 2, *H. alternicirrhosum* and *H. ginfushanicum*.

Given that codon usage is closely related to genome-wide protein and mRNA levels, it is an essential feature of gene expression. The same codon presents different frequencies in different organisms. The codon usage frequencies of *Polygonatum campanulatum*, *P. filipes*1, *P. franchetii*, *P. zanlanscianense*1, *P. cyrtonema*1, *P. sibiricum*1*, P. kingianum*2, *Heteropolygonatum alternicirrhosum* and *H. ginfushanicum* were computed based on protein-coding genes of the complete chloroplast genome. The total codons in these nine species varied from 26,453 codons (*P. kingianum*2) to 26,651 codons (*P. zanlanscianense*1). The most abundant amino acid (AA) was leucine (Leu), with the proportions ranging between 10.2 and 10.3%, followed by serine (Ser) accounting for 7.8–7.9% (Table S3)*.* In contrast, cystine (Cys) possessed the lowest number of codons (306–309 codons) in all the nine species when terminal codons were not considered. The AGA codon, encoding arginine (Arg), presented the highest RSCU (relative synonymous codon usage) value of 10.92–1.96, while AGC codon, encoding serine (Ser), showed the lowest RSCU value with 0.31–0.33 (Table S3). Additionally, CGC encoding Arginine (Arg) and AGC encoding serine (Ser) shared the lowest RSCU value of 0.31–0.32 and 0.31–0.33 respectively. Figure 2 illustrates the summary statistics for amino acid frequency and relative synonymous codon usage. Among the 64 codons, there were 31 codons with RSCU values less than 1 (RSCU < 1), which showed a lower usage frequency than expected. Meanwhile, 30 codons were used more frequently than expected in *P. campanulatum* and *P. filipes*1 with RSCU values greater than 1 (RSCU > 1), while 31 codons in the other seven species. Furthermore, the RSCU values of AUG and UGG in all the nine species were equal to one (RSCU = 1) appearing without usage preference, while UCC only showed the same characteristics in *P. campanulatum* and *P. filipes*1. Particularly, methionine (AUG) and tryptophan (UGG) were encoded by only one codon. All codons with RSCU > 1 were characterized by Adenine–Thymine ending in the six species apart from UUG and the UCC in *P. franchetii, P. zanlanscianense*1, *P. cyrtonema*1, *P. sibiricum*1*, P. kingianum*2, *H. alternicirrhosum* and *H. ginfushanicum*. On the contrary, 28 of the 31 codons with RSCU < 1 were detected ending with Guanine-Cytosine (GC) in each species. When comparing nine *Polygonatum*, there were nearly no differences in RSCU value, indicating that the codon use bias of *Polygonatum* is rather stable (Fig. 2).

### Long dispersed repeats and microsatellites analysis

**Figure 3:**
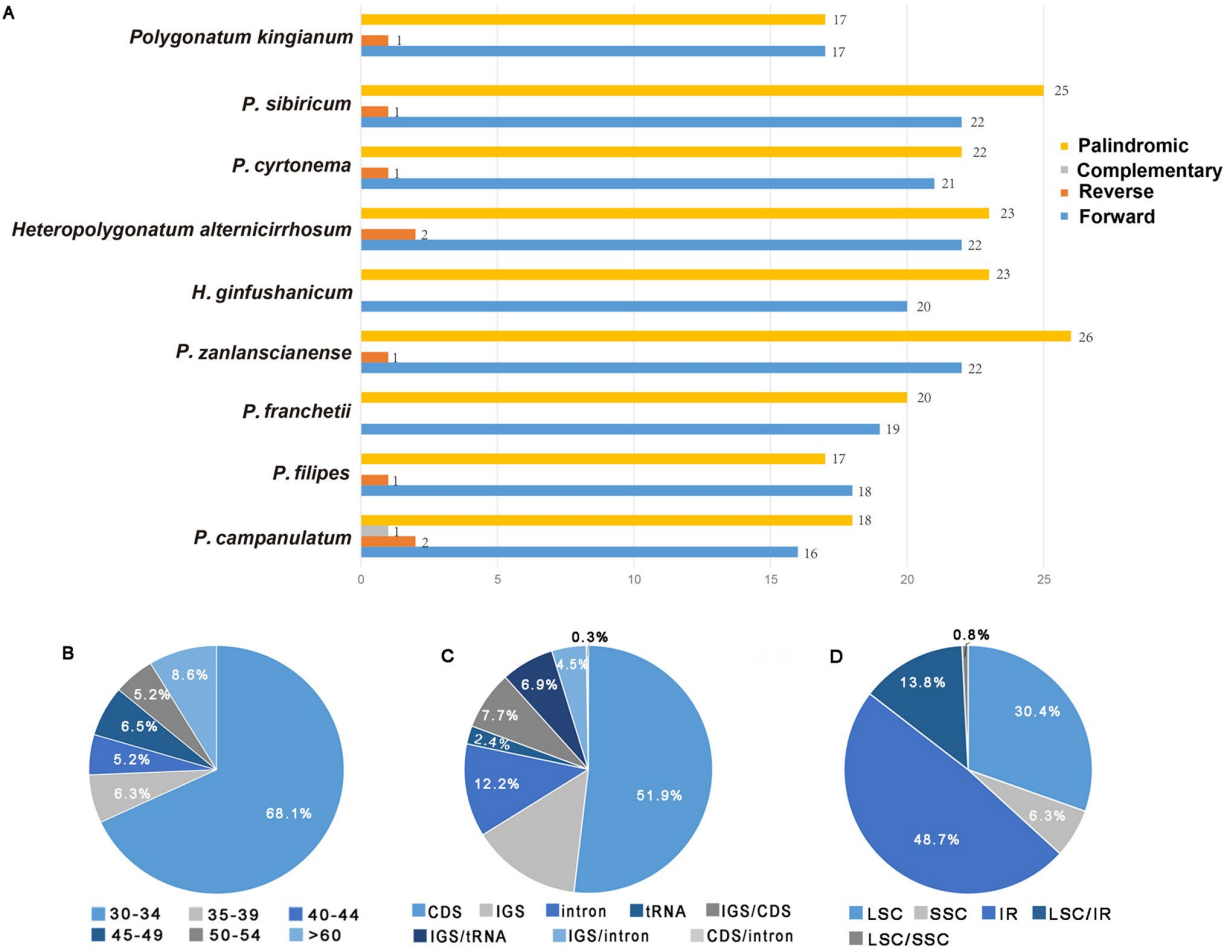
Analysis of long dispersed repeats in the cp genomes of seven *Polygonatum* and two *Heteropolygonatum* species. (**A**) The number of the four types of long repeats. (**B**) Distribution ratio of repeats in regions of the cp genome. (**C**) Distribution ratio of repetitive sequences in functional regions. (**D**) Proportion of repeats in different length intervals of the chloroplast genome.

A total of 378 long dispersed repeats were observed in the seven *Polygonatum* and two *Heteropolygonatum* species, consisting of 191 palindromic repeats, 177 forward repeats, nine reverse repeats and one complementary repeat (the palindromic repeat of IR regions itself was excluded in all the nine species) (Table S4). Obviously, palindromic repeats were the dominant repeat type (from 47.2% in *P. filipes*1 to 53.5% in *P. zanlanscianense*1), while complementary repeats were the least frequent one which was only detected in *P. campanulatum* (2.7%). Likewise, *P. franchetii* and *H. ginfushanicum* did not possess any reverse repeats. On the other hand, the species that harbor the highest number of long repeats was *P. zanlanscianense*1 (49), and the species with the lowest number was *P. kingianum*2 (35) (Fig. 3A). In *H. ginfushanicum*, the length of the longest repeat sequence was 66 bp while in the rest eight species were 71 bp, and all of them were forward repeats. Furthermore, among all repeats detected in the nine species, the length of repeats ranging from 30 to 34 bp accounted for the majority (260, 68.1%) (Fig. 3B, Table S5). The most repeats were detected in the CDS, followed by IGS regions, some repeats were also identified between CDS, IGS, tRNA and introns (Fig. 3C, Table S6). Most of the repeat sequences were located in the IR regions except for *P. campanulatum* and *P. filipes*1, which harbored the highest number of repeats in LSC region (Fig. 3D, Table S7).

**Figure 4:**
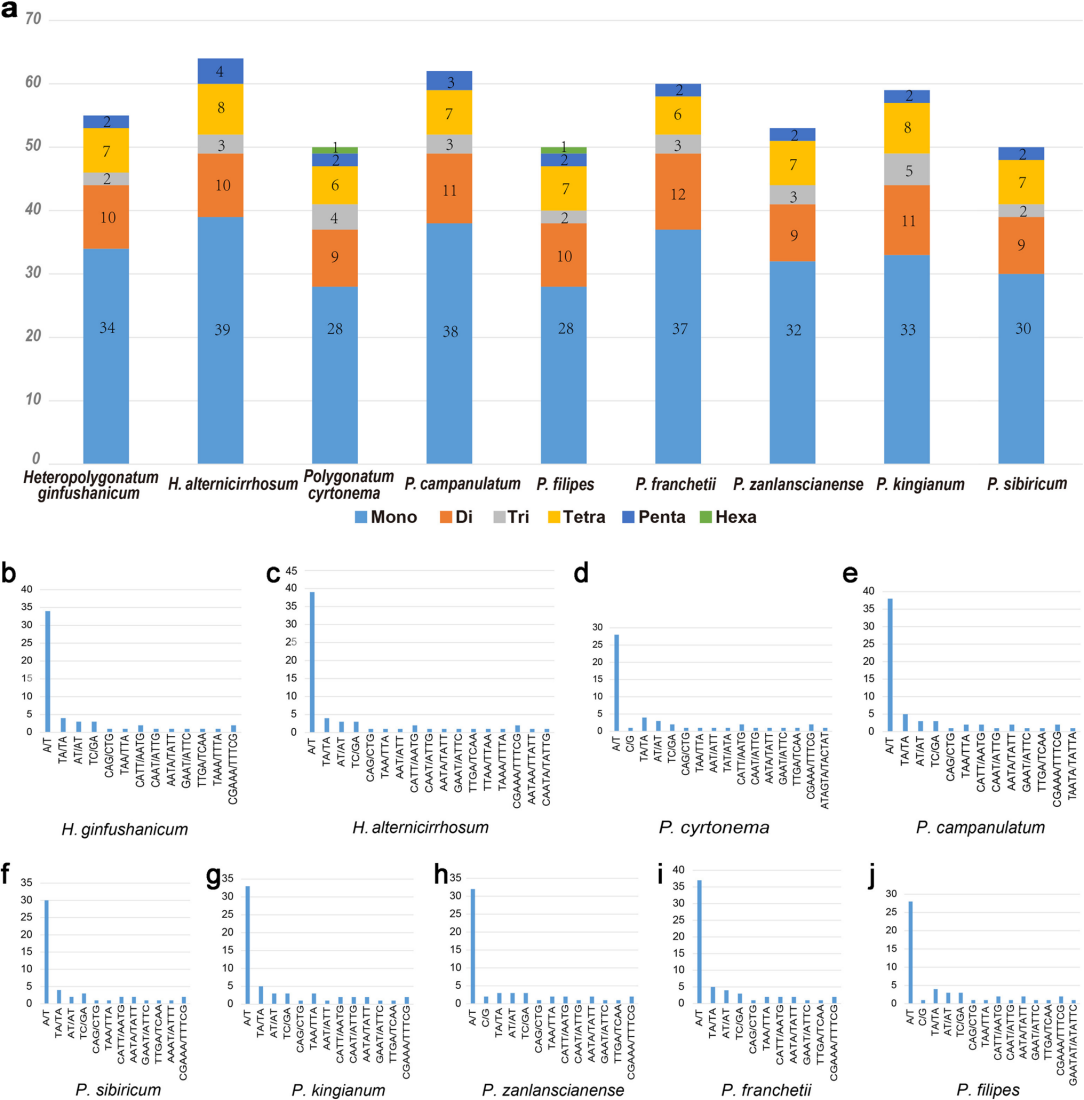
Simple sequence repeats (SSRs) analysis of the complete chloroplast genomes of the seven *Polygonatum* and two *Heteropolygonatum* species. (**a**) Numbers of mono-, di-, tri-, tetra-, penta-, and hexa-nucleotide repeats. (**b–j**). Frequencies of SSRs motifs in different repeat class types.

In this study, we observed 507 SSRs among the nine species in total, comprising 303 mono-, 91 di-, 27 tri-, 63 tetra-, 20 penta-, and two hexa-nucleotide repeats (Table S8). Moreover, a total of two mono-, three di-, four tri-, eight tetra-, four penta-types and two hexa-nucleotide repeats types were identified. And one tri-, two tetra-, three penta- and two hexa-nucleotide types were observed only once in only one species (Table S9). Most SSRs were mononucleotide and dinucleotide repeats, besides, the rest of SSRs showed lower frequencies. As shown in Fig. 4a, mono-nucleotide repeats were the most frequent type ranging from 55.9% (*Polygonatum kingianum*2) to 61.8% (*Heteropolygonatum ginfushanicum*). The number of SSRs of *H. alternicirrhosum* reached a peak value of 64 among the nine species. On the other hand, *P. sibiricum*1 possessed the least number of SSRs of 50 (Fig. 4a, Table S9). The most dominant SSRs were A/T polymers (Fig. 4b–j), suggesting a remarkable base preference. And the majority of the microsatellites were located in the LSC region (Table S10). These results indicate that there were no distinctive differences in SSRs between *Polygonatum* and *Heteropolygonatum*. The identified SSRs will provide valuable genetic information for the phylogeny and population genetics of *Polygonatum* in the future.

### Comparative genome analysis and sequence variation

**Figure 5:**
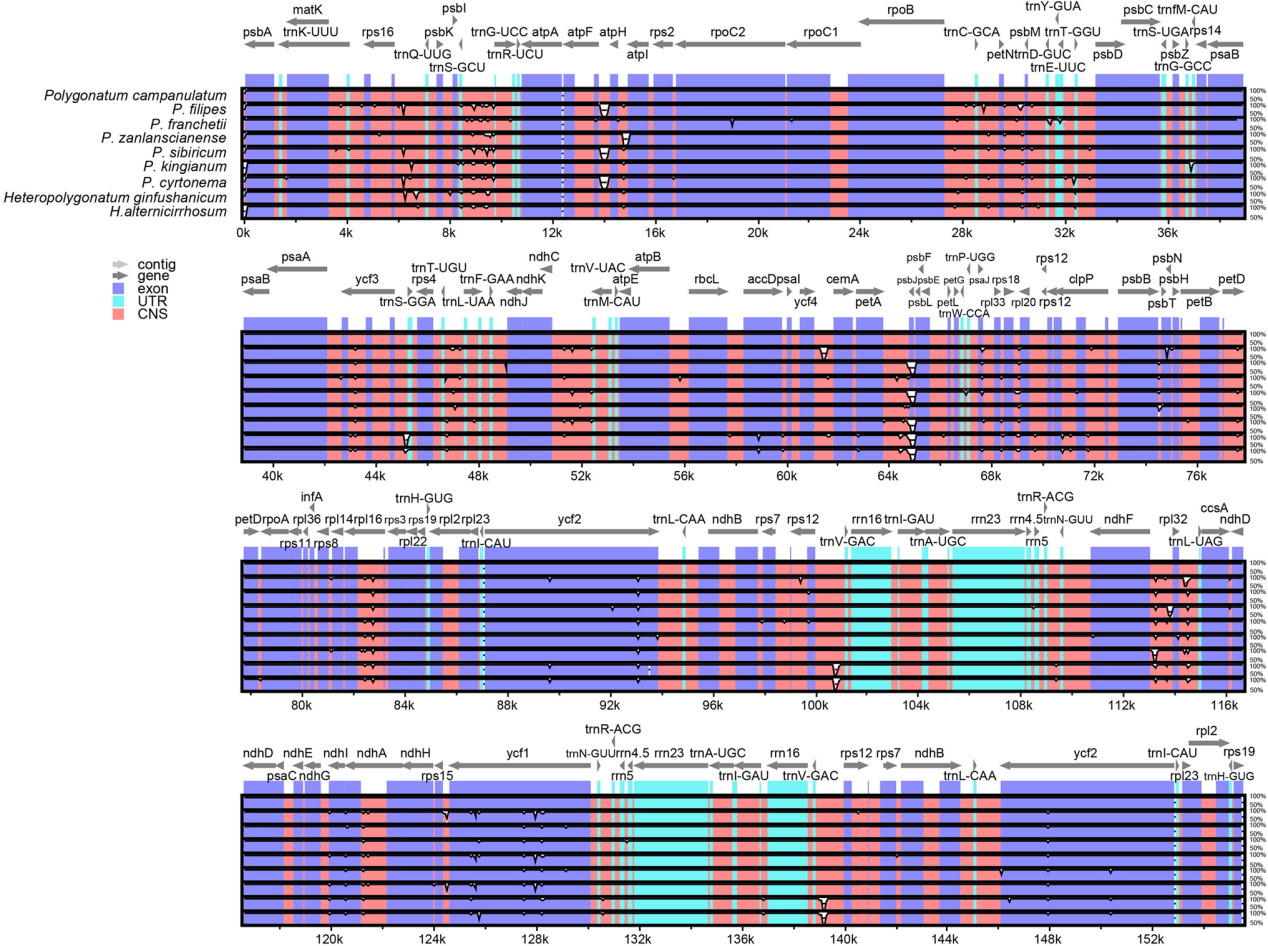
Alignment of chloroplast genomes of *Heteropolygonatum alternicirrhosum*, *H. ginfushanicum*, *Polygonatum campanulatum*, *P. filipes*1, *P. franchetii*, *P. zanlanscianense*1, *P. cyrtonema*1, *P. sibiricum*1, *P. kingianum*2. The grey arrows at the top represent the direction of gene translation, and the y-axis indicates the percentage identity between 50 and 100%. (Exon: protein codes; UTR: tRNAs and rRNAs; CNS: conserved noncoding sequences).

**Figure 6:**
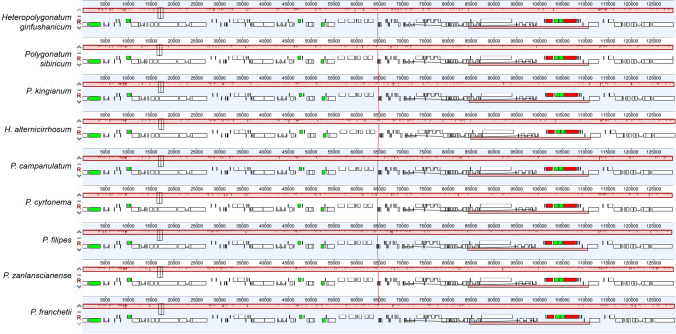
Genomic rearrangement of the seven *Polygonatum* and two *Heteropolygonatum*. Blocks in different colors correspond to different gene types. Black: transfer RNA (tRNA); green: intron-containing Trna; Red: ribosomal RNA; White: protein-coding genes (PCGs).

To identify highly variable regions among the seven species of *Polygonatum* and two species of *Heteropolygonatum,* multiple sequence alignment of the cp genomes was carried out. The annotation of *Polygonatum campanulatum* was set as a reference. It can be seen from the data in Fig. 5 that coding regions were much more conserved than non-coding regions, with almost no significant variations except for *ycf1*. Additionally, we detected that some intergenic spacer region and introns appeared considerable variations, including *rps16*-*trnQ*, *trnS*-*trnG*, *atpF*-*atpH*, *atpH*-*atpI*, *petA*-*psbJ*, *ndhF*-*rpl32*, *rpl32*-*trnL and rpl16*. Another significant result was that compared with the IRs regions, LSC and SSC regions showed higher variation, consistent with the result of nucleotide polymorphisms analysis (Fig. 8). Apart from *ycf1*, all highly divergent regions mentioned above were in single-copy regions. With respect to tRNA and rRNA, they were strongly conserved without evident variations. Additionally, collinearity detection analysis found that there were no interspecific or intraspecific rearrangements in the nine species (Fig. 6).

### Expansion and contraction of IRs

**Figure 7:**
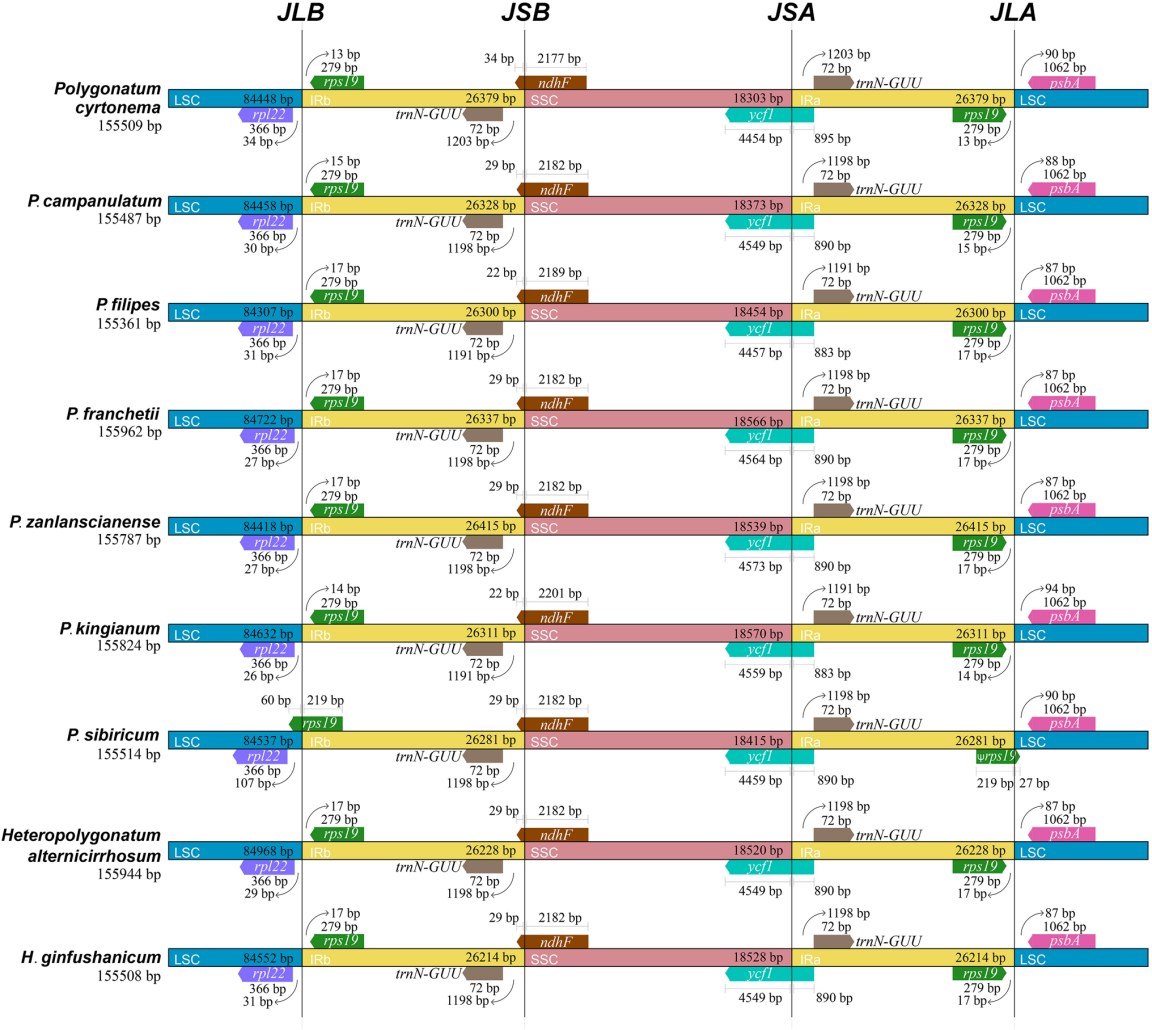
Comparative analysis of the LSC, IR and SSC boundary regions in the nine chloroplast genomes.

A comprehensive comparison of boundaries between single-copy and the IRs regions was carried out. We observed that the complete cp genome structure of the nine species varied from each other slightly. Apart from *Polgonatum sibiricum*, the junctions of LSC/IRb sit between *rpl22* gene and *rps19* gene among the other eight species. The *rpl22* gene was located in the LSC region completely with 26 bp to 34 bp away from LSC/IRb border, while the *rps19* genes within IR regions were close to two IR/LSC boundaries. Furthermore, in *P. sibiricum*, two *rps19* genes extended into the LSC region due to the contraction of IRs (Fig. 7), leading to the one located at IRa/LSC junction being a pseudogene. Apart from this special case, *rps19* in the other species was quite conservative with the same length of 279 bp. Likewise, *rpl22* gene was also very conserved with the same length of 366 bp in all the nine species. Moreover, the *ndhF* gene was located in the boundaries of IRb/ SSC and expanded to the IRb region by 22, 29, or 34 bp. And *trnN* gene was close to the IRs/SSC boundaries with the whole gene within IRs regions. The *ycf1* gene ranges from 4454 to 4573 bp and straddled the SSC/IRa boundary, with 883–895 bp distributed in the IRa region and the rest in the SSC region (Fig. 7). In terms of IRa-LSC boundary, *rps19* gene was located on the left side while *psbA* gene was on the right, and *psbA* gene was highly conserved with a steady length of 1062 bp. The distances between *psbA* and the IRa/LSC junction varied from 87 to 94 bp.

Together these results provided important insights into contractions and expansions of IR region borders *in Polygonatum and Heteropolygonatum.* The structures and gene orders of the two genera were relatively conserved except for *P. sibiricum*, in which a slight expansion and contraction occurred between IRs and LSC.

### Nucleotide diversity and selective pressure analysis

**Figure 8:**
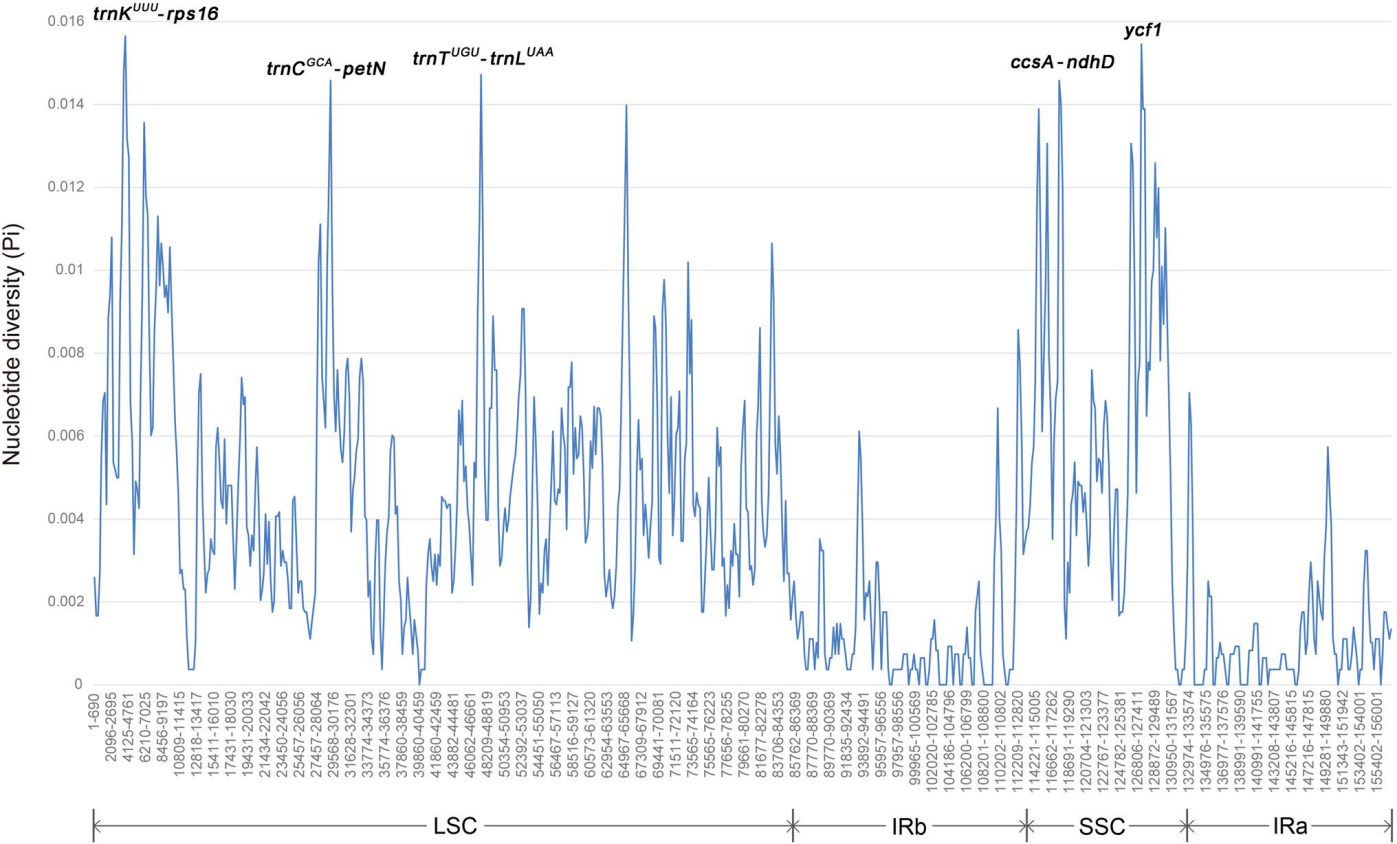
Nucleotide diversity analysis of the complete chloroplast genomes of the seven *Polygonatum* and two *Heteropolygonatum* (window length: 600 bp; step size: 200 bp).

The nucleotide diversity of nine chloroplast genomes of *Polygonatum* and *Heteropolygonatum* was calculated to detect divergence hotspots. The pair of inverted repeats were relatively conserved regions with an average Pi value of 0.00113. At the same time, LSC and SSC showed higher nucleotide diversity with a mean Pi value of 0.00492 and 0.00674 respectively. Significant variations (Pi > 0.014) were found in the following regions: *trnK*^*-UUU*^-*rps16*, *trnC*^*-GCA*^-*petN*, *trnT*^*-UGU*^-*trnL*^*-UAA*^, *ccsA*-*ndhD* and *ycf1* (Fig. 8), in which the most divergent region was *trnK*^*-UUU*^-*rps16*, with the Pi value of 0.01565. Of these five regions, 80% (4) were intergenic genes. In contrast, protein-coding regions accounted for 20% (1), indicating that non-coding regions harbored more variations and coding region were more stable and conservative. Moreover, all five divergent hotspots might be potential molecular markers for DNA barcodes adopted into species identification and phylogenetic studies in the future.

Synonymous substitutions in the nucleotide preserve the same amino acids. On the contrary, non-synonymous substitutions will change the amino acids. The substitution rates of nonsynonymous (dN) and synonymous (dS) have been widely used for quantifying adaptive molecular evolution in the chloroplast genome^50^. In the current study, according to BEB methods, a total of 14 genes corresponding to 65 sites were detected under positive selection. Among them, four genes (*rpoC2*, *rpoB*, *psaA*, *ndhK*) were identified under significant positive selection, and ten genes (*psbA*, *psbK*, *atpA*, *rpoC1*, *psbD*, *psbC*, *psbZ*, *psaB*, *rps4*, *ndhJ*) under positive selection (Table S11). All the selected genes were located in LSC regions, and 10 were related to photosynthesis. We observed that *rpoC2* harbored the highest number of sites under positive selection (13), followed by *psaA* (12) and *rpoB* (11).

### Phylogenetic analysis of *Polygonatum*

**Figure 9:**
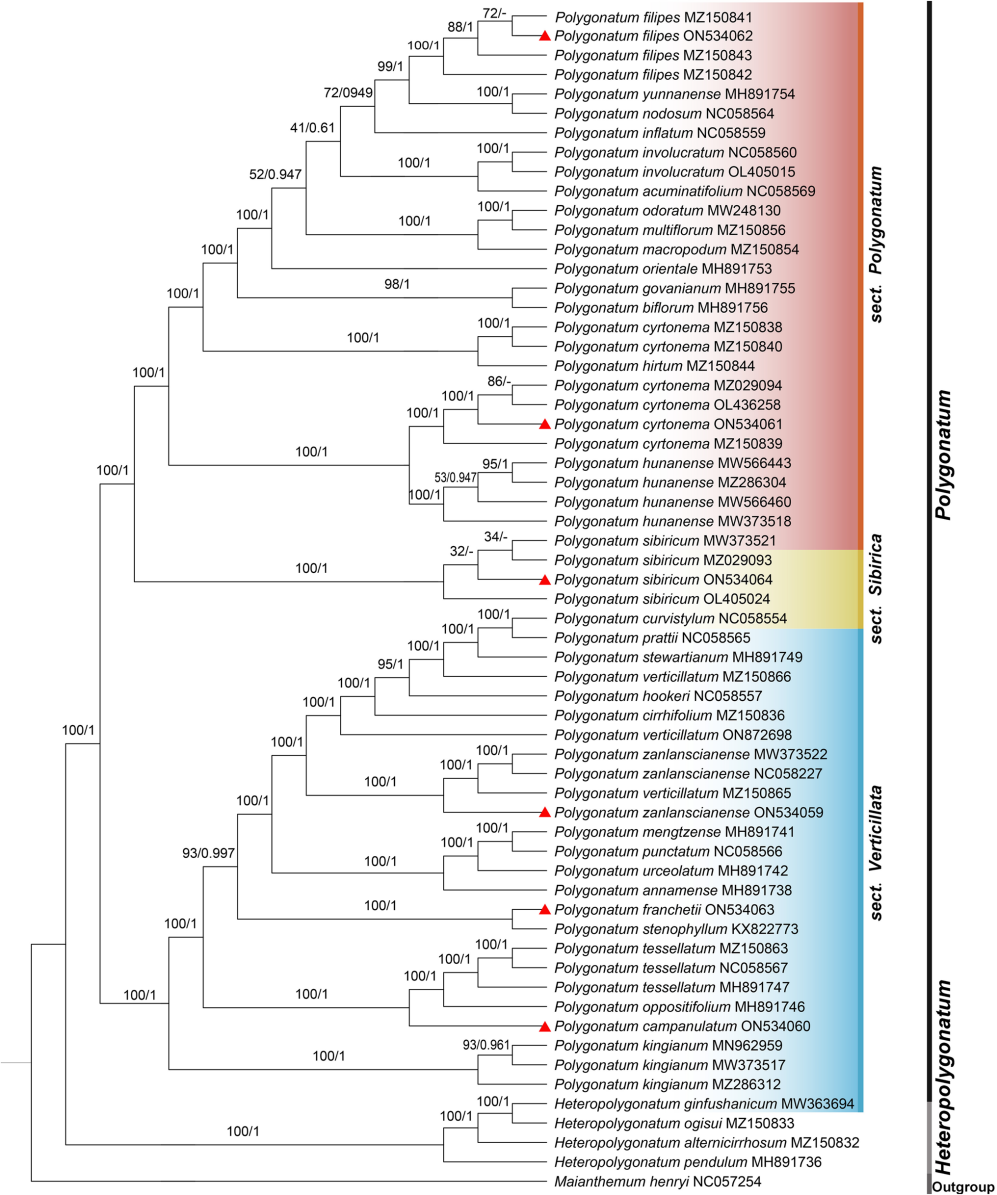
Phylogenetic relationships of the 57 cp sequences of *Polygonatum* and 4 of *Heteropolygonatum*, with *Maianthemum henryi* set as the outgroup. Maximum likelihood (ML) and Bayesian inference (BI) methods were used to reconstruct the tree. Only ML tree was shown, because of the highly identified topologies of ML tree and BI tree. The value of ML supports and Bayesian posterior probabilities were shown above the branches. The cp genomes newly sequenced in this study are highlighted with red triangle marks.

A total of 62 cp sequences of *Polygonatum* and its related species were selected to reconstruct phylogenetic relationships among this genus. *Maianthemum henryi* was chosen as an outgroup own to its closer distances and more basic position to *Polygonatum* and *Heteropolygonatum*. The 62 cp sequences comprise six newly sequenced data (i.e., *Polygonatum campanulatum*, *P. filipes*1, *P. franchetii*, *P. zanlanscianense*1, *P. cyrtonema*1, *P. sibiricum*1) and 56 cp genome published in NCBI (Table S1). The topologies of Maximum likelihood (ML) and Bayesian inference (BI) were highly identical both in tree structure and species position with generally strong support (Fig. 9). The difference lies in the fact that the BI analysis cannot tell apart the branch structure of some different samples belonging to the same species (Fig. 9). Both *Polygonatum* and *Heteropolygonatum* exhibited monophyletic relationships and shared the most recent common ancestor. *Polygonatum* was divided into two main lineages including sect. *Verticillata* and the clade consisting of sect. *Polygonatum* and sect. *Sibirica*. Phylogenetic analysis suggested that sect. *Sibirica* comprise only one species, i.e., *P. sibiricum*. Moreover, we also observed that *P. verticillatum* and *P. cyrtonema* were paraphyletic. *P. verticillatum*1 was sister to *P. zanlanscianense* (BS = 100, PP = 1.00), while *P. verticillatum*2 appeared as sister clade to *P. curvistylum* + *P. pratti* + *P. stewartianum* (BS = 100, PP = 1.00), and *P. verticillatum*3 located at the base of the branch composed by *P. curvistylum* + *P. pratti* + *P. stewartianum* + *P. verticillatum*2 + *P. hookeri* + *P. cirrhifolium* + *P. verticillatum*3 (BS = 100, PP = 1.00). Four samples of *P. cyrtonema,* including the newly sequenced one, appeared as the sister to* P. hunanense* (BS = 100, PP = 1.00) and this clade locates at the base of sect. *Polygonatum*. The other two samples present as sister clade to *P. hirtum* with significantly high Bayesian posterior probability and bootstrap support (BS = 100, PP = 1.00). For *P. franchetii,* it was the sister clade to *P. stenophyllum* (BS = 100, PP = 1.00)*.* Furthermore, *P. filipes* strongly supported being included in sect. *Polygonatum* and being sister to *P. yunnanense* plus *P. nodosum* (BS = 99, PP = 1.00). Surprisingly, *P. campanulatum* with alternate leaves located in sect. *Verticillata*, a group characterized by whorled leaves, and formed a sister clade with *Polygonatum tessellatum* plus *Polygonatum oppositifolium* (BS = 100, PP = 1.00), which suggested that leaf arrangement is not suitable as the basis for delimitation of subgeneric groups in *Polygonatum*.

## Discussion

### Features of complete chloroplast genome and comparative analyses

In the current study, we reported the initial complete cp genomes for one critically endangered *Polygonatum* species, *Polygonatum campanulatum.* Additionally, the complete cp genomes of other five species were newly sequenced (*P. cyrtonema*1*, P. franchetii, P. filipes*1*, P. zanlanscianense*1*, P. sibiricum*1) using Illumina sequencing technology. Besides, cp genomic comparative analyses of the plastomes were carried out among the six species plus another three related species (*P. kingianum*2, *Heteropolygonatum alternicirrhosum*, *H. ginfushanicum*) to understand potential genetic information of *Polygonatum*. The cp genome showed a typical quadripartite structure, with the length between 154,564 and 156,028 bp in *Polygonatum*, and 155,436–155,944 bp in *Heteropolygonatum.* The range of chloroplast genome length variation in these two species was similar to other Asparagaceae and higher plants reported previously^51–55^. And the size changes are partially caused by elongation or contraction of inverted repeat regions.

Our study revealed that gene content and gene order in the cp genomes of *Polygonatum* and *Heteropolygonatum* were highly conserved, with only slight variations in gene size, gene position and gene number. This result is similar to other species of Asparagaceae^56^. All plastomes contained 131–132 genes comprising 85–86 protein-coding genes, 38 tRNA and eight rRNA. Among these genes, 18 included intron and 19 were duplicated in IR regions. The difference in gene number is due to pseudogenization of *rps19* and *ycf1* in some sequences. In detail, one of the *rps19* genes in *P. stewartianum*, *P. sibiricum*1 and *P. sibiricum*2 presented to be a pseudogene. The first one is attributed to genetic mutation and the others to its location at IR/LSC boundary, which makes the gene lose its ability to replicate fully. And, both *ycf1* genes were detected pseudogenized in *H. ogisui* due to the insertion of a sequence Expression of the *rps19* gene is relatively unstable among species of Asparagaceae, the pseudogenization of *rps19* has also been reported in *Behnia reticulate, Hesperaloe parviflora* and *Hosta ventricosa*, while *Camassia scilloides* and *Chlorophytum rhizopendulum* missed this gene completely^57^. The *rps2*, *infA* and other pseudogenes reported previously in Asparagaceae were not detected in this study^57,58^. In addition, although there were no remarkable variations in GC content among different species, the distribution of GC content was identified as asymmetrical. The higher GC content in IRs means a more stable structure in that GC pairs include three hydrogen bonds and AT pairs have two^59^. Moreover, this may be attributed to the four rRNA genes, which possess high-level GC nucleotide percentages. Similar results have been found in the chloroplast genomes of other angiosperms^60–62^.

The pattern of codon usage is a vital genetic characteristic of the organism, related to mutation, selection and other molecular evolutionary phenomena^63^. Our results demonstrated that Leucine (Leu) presented the highest frequency of all amino acids in *Polygonatum campanulatum*, *P. filipes*1, *P. franchetii, P. zanlanscianense*1*, P. cyrtonema*1, *P. sibiricum*1*, P. kingianum*2, *Heteropolygonatum alternicirrhosum* and *H. ginfushanicum*. On the contrary, cystine (Cys) was the least abundant amino acid except for stop codons, which was also found in other angiosperm taxa^24,64^. Furthermore, The result of RSCU analysis illustrated that most codons ended with A or U when RSCU value was greater than one, likewise, most codons ended with C or G when the RSCU value was less than one. This phenomenon revealed that codon usage was biased towards A and U at the third codon position in *Polygonatum*, which coincided with previous studies^56,61,65^.

Long dispersed repeats are essential for the rearrangement and stability of the chloroplast genome and relevant to copy number differences among species^66^. Identifying their number and distribution plays a key role in genomic studies^67^. The current study found that palindromic repeats were the most common repeat type, followed by forward repeats. Whereas complementary repeat was identified only in *P. campanulatum*, *P. franchetii* and *H. ginfushanicum* did not harbor any reverse repeats. In the plastomes of the nine species reported here, the length of repeats ranging from 30 to 39 bp is dominant, which is commonly observed in other angiosperm lineages^31,52,68^**.** Our study also revealed that the repetitive sequences were not randomly allocated in the seven cp genomes of *Polygonatum* and two cp genomes of *Heteropolygonatum*, they were mainly identified in the LSC region (48.7%) and CDs (51.9%).

SSR (Simple Sequence Repeats) is a significant codominant DNA molecular marker with the advantages of high abundance, random distribution throughout the genome and ample polymorphism information^69,70^. Therefore, it provides essential insights into many fields, such as species identification, phylogeography and population genetics^71,72^. A total of 507 SSRs were detected in the current study, with *H. alternicirrhosum* containing the most. Further, among the seven cp genomes of *Polygonatum* and two cp genomes of *Heteropolygonatum*, six categories of SSRs were observed in total. Mononucleotide SSRs showed the highest frequency in each genome, with A/T as the predominant motif type. Similar results had been reported in numerous taxa^53,61,73^. By contrast, hexanucleotide SSRs were the rarest type, with only one element being observed in *P. cyrtonema*1 and *P. filipes*1. In addition, SSRs lying within LSC regions accounted for the majority (72.4%), which was in agreement with previous studies^65,68^. In summary, the microsatellites identified in this study will be developed as markers for *Polygonatum*, and contribute to species identification and evolutionary studies of this genus in the future.

Multiple sequence alignment results revealed the similarities of cp genome in structure, content, and order among *Polygonatum* and its related species. Consistent with previous reports^74–76^, we also found that no coding regions harbored more distinctive variation than coding regions in this study. Two single-copy regions exhibited higher sequence divergence than the IRs. The following seven intergenic regions, i.e., *rps16*-*trnQ*, *trnS*-*trnG*, *atpF*-*atpH*, *atpH*-*atpI*, *petA*-*psbJ*, *ndhF*-*rpl32*, *rpl32*-*trnL* and two genes, i.e., *ycf1 and rpl16* were detected as the most divergent. Comparative analysis of *Polygonatum* and its related species discovered that the cp genomes presented highly conserved, and no interspecific or intraspecific rearrangement was detected.

Contraction and expansion in IRs regions led to variations in cp genome size, which were observed in the evolutionary history of terrestrial plants commonly^62^. The size of IR regions was relatively similar in *Polygonatum* and *Heteropolygonatum*, ranging from 26,214 bp in *H. ginfushanicum* to 26,415 bp in *P. zanlanscianense*1. Despite that, all the cp genomes showed similarity in the overall gene order and structures, several variations were identified at the junctions of IR/SC. The current study demonstrated that boundary genes in *Polygonatum* were mainly *rpl22*, *rps19*, *trnN*, *ndhF*, *ycf1* and *psbA,* which is also identified with *Heteropolygonatum* and *Hosta*^56^. It further confirms that boundary features are relatively stable across closely related species^77^. The LSC/IRb boundary was traversed by the *rps19* gene in *P. sibiricum*1*,* whereas the junctions located between *rpl22* and *rps19* in the other species. Incomplete duplication of the normal copy resulting in pseudogenization of the *rps19* gene located at IRa/LSC boundary, and this phenomenon has also been reported in *Polygonatum cyrtonema* (MZ029094)^14^ and other taxa of Asparagaceae, such as *Behnia reticulate, Hesperaloe parviflora* and *Hosta ventricosa*^57^. Excluding *rps19*, the other genes situated at SC/IR boundaries exhibited relative stability across the six *Polygonatum* and two *Heteropolygonatum* species studied in this work. Only *ndhF* and *ycf1* had slight variations in size. The high resemblances in boundaries between SC/IR also demonstrate that all the species share the same genes. Besides, the total number of genes does not change due to IR contraction and expansion^78^.

We detected *trnK*^*-UUU*^-*rps16*, *trnC*^*-GCA*^-*petN*, *trnT*^*-UGU*^-*trnL*^*-UAA*^, *ccsA*-*ndhD* and *ycf1* were prominent divergent regions, with nucleotide diversity greater than 0.014. There are three loci (*matK-rps16*, *trnC*^*-GCA*^-*petN* and *ccsA*) consistent with previous study^14^. The result indicated that divergent regions located in LSC were in the majority, and the IR regions displayed relatively poor diversity, which agreed with the results of multiple sequence alignment conducted by mVISTA. The same phenomenon has been observed in many taxa^24,31^. The regions detected in nucleotide diversity analysis might also provide additional genetic information for DNA barcodes in *Polygonatum*, but this required the support of further experiments.

The non-synonymous (dN) and synonymous (dS) substitution rates are beneficial in inferring the adaptive evolution of genes^25,79^. The analysis of dN/dS was carried out owing to its popularity and reliability in quantifying selective pressure^80,81^. In this study, a total of 14 positively selected sites (comprising 4 significant positive and 10 positive sites) were detected under the BEB method, which were distributed in *atpA, ndhJ, ndhK*, *psaA*, *psaB*, *psbA*, *psbC, psbD, psbK, psbZ, rpoB, rpoC1, rpoC2, rps4.* Results indicated that 10 of the 14 positively selected genes are relevant to photosynthesis (Table S11). The plants of *Polygonatum* are mainly distributed in the shady places of forest, scrub or mountain slopes^11^. The week sunlight may exert selective pressure on genes, which could leave a trace of natural selection in genes of chloroplast engaged in adaptation to the environment. It can be speculated that photosynthesis-related genes drive the successful adaptation of *Polygonatum* to diverse environment conditions, considering their extensive distribution range in the northern hemisphere. Photosynthesis-related genes were also found to undergo positive selection in other taxa that are widely distributed or live in shady environments^82–86^.

### Phylogenetic analysis

Phylogenetic analysis based on complete cp genome demonstrated that both *Polygonautm* and *Heteropolygonatum* were monophyly. Coinciding with the results of previous studies^7,13,28^, *Polygonatum* was composed of three major clades, sect. *Verticillata,* sect. *Sibirica* and its sister clade sect. *Polygonatum*. In the current study, we observed that sect. *Sibirica* contained only one species, *P. sibiricum*, which was consistent with Xia, Meng and Wang’s findings^7,14,28^. However, data from Floden^13^ suggests that one sample of *P. verticillatum* was sister to *P. sibicirum* within sect. *Sibirica*. Moreover, previous studies indicated that *P. verticillatum* was paraphyletic, potentially as a result of its wide geographic distribution and diverse morphological variations^13,28^. A similar result was presented in this study. *P. verticillatum*1 exhibited as the sister clade to *P. zanlanscianense* while *P. verticillatum*2 was sister to *P. curvistylum* + *P. pratti* + *P. stewartianum,* and *P. verticillatum*3 located at the base of the branch composed by *P. curvistylum* + *P. pratti* + *P. stewartianum* + *P. verticillatum*2 + *P. hookeri* + *P. cirrhifolium* + *P. verticillatum*3. With similarities to previous findings^28^,* P. cyrtonema* was either recovered as paraphyletic in this study given that four samples, including the newly sequenced one, appeared as the sister to* P. hunanense*, while the other two samples presented being sister relationship with *P. hirtum*. All the clades were supported highly. It suggests that the circumscription of these two broadly distributed species, *P. cyrtonema* and *P. verticillatum* requires further study.

There is little study on the systematic position of *P. franchetii*, and even less on the its cp genome information. Meng’s team^7^ reported the phylogenetic relationships included in *P. franchetii* using four chloroplast fragments (*rbcL*, *psbA-trnH*, *trnK* and *trnC-petN*) for the first time. Regrettably, the branch structure to which *P. franchetii* belonged was ambiguous, making it difficult to recognize the relationship between *P. franchetii* and its close taxa. Wang-Jing^14^ reported the cp genome of *P. franchetii* for the first time. However, the sample chuster with *P. hirtum* + *P. multiflorum* and located in *sect. Polygonatum*, which shows difference with this study. Our study suggests that *P. franchetii* is strongly supported as the sister clade to* P. stenophyllum* and is situated in *sect. Verticillata*. Furthermore, *P. filipes* presented the sister clade to *P. yunnanense* plus *P. nodosum* within sect. *Polygonatum* in this study*.* And it is found by Xia et al.^28^ that *P. filipes* was the sister to the clade consisting of *P. inflatum* + *P. multiflorum* + *P. odoratum* + *P. macropodum* + *P. involucratum* + *P. acuminatifolium* + *P. arisanense* + *P. orientale* + *P. yunnanense* + *P. nodosum* with high support. However, the clade composed of *P. yunnanense* + *P. nodosum* was weakly supported as the sister to the rest species in the sister clade of *P. filipes.* Besides, *P. filipes* is the sister clade to *P. cyrtonema,* and this branch clusters with *P. jinzhaiense* and *P. hunanense* in Wang-Jing’s study^14^. It suggests that the voucher specimens of *P. filipes* and *P. franchetii* in Wang’s study^14^ should be checked further.

One unanticipated finding was that phylogenetic tree strongly supported the placement of *Polygonatum campanulatum* in sect. *Verticillata,* despite the fact that *P. campanulatum* grows alternating leaves, but sect *Verticillata* is characterized by whorled or opposite leaves. *P. campanulatum* was compared to *P. gongshanense* and *P. franchetii* when it was first published, but material for *P. gongshanense* was not available in this work. Furthermore, phylogenetic analysis indicated that *P. franchetii* and *P. campanulatum* presented in separate branches whereas *P. tessellatum* + *P. oppositifolium* were highly supported as the sister to *P. campanulatum* (BS = 100, PP = 1.00). Despite *P. campanulatum*, *P. tessellatum* and *P. oppositifolium* sharing similar lustrous and lanceolate leaves^2,87^, they differ in leaf arrangement, filament structure and florescence, etc. In detail, *P. campanulatum* is characterized by alternate leaves with a retrorse spur at the filament apex and flowers in October, while *P. tessellatum* and *P. oppositifolium* differ in whorled or opposite leaves without a retrorse spur at the filament apex and flower in May^2,87^. Moreover, previous studies discovered that leaf arrangement is labile and the whorled leaves have arisen from the alternate state at least twice^7,88^. In conclusion, we infer that the use of phyllotaxis to define subgenera within *Polygonatum* is inappropriate. Additionally, blossom color and pollen exine sculpture were also used as the features to subgroup *Polygonatum* in previous studies^7,12,89^. Whereas sect. *Verticillata* typically displayed reticulate pollen exines and purple or pink perianths, sect. *Polygonatum* was distinguished by its perforated pollen exines and greenish-white or yellow perianths^7,89^. In contrast, *P. campanulatum* placed in *Verticillata* has perforate reticulate decorations and perianths that are either yellowish green or greenish white^87^. The controversy over flower color has been reported in the study of Xia and her team^28^. From this, we can see that flower color and pollen exine sculpture may be irrelated with phylogeny and not ideal as the basis for subgenus classification of *Polygonatum* either. Moreover, further research about the information is required on base chromosome numbers and karyotypes of *P. campanulatum*. This work will contribute to a more insightful understanding of the infrageneric classification of *Polygonatum* and demonstrate that the cp genome is an efficient tool for resolving specific level phylogeny.

## Conclusion

In the current study, we sequenced and annotated the cp genomes of *Polygonatum campanulatum*, *P. franchetii*, *P. filipes*1, *P. zanlanscianense*1, *P. cyrtonema*1 and *P. sibiricum*1*.* Comparative analyses of the chloroplast genome of the six taxa and three related species were conducted. The genome size, gene content, gene order and G-C content maintained a high similarity in the cp genomes of *Polygonatum* and *Heteropolygonatum*. No interspecific or intraspecific rearrangements were detected. Five highly variable regions were found to be potential specific DNA barcodes. Fourteen genes were revealed under positive selection and a large variety of repetitive sequences were identified. Sixty-two cp sequences of *Polygonatum* and its related species were utilized for phylogenetic analyses. The phylogenetic results illustrated that *Polygonatum* can be divided into two significant clades, sect. *Verticillata* and sect. *Sibirica* plus sect. *Polygonatum.* Further, *P. campanulatum* and *P. tessellatum* + *P. oppositifolium* were strongly supported being sister relationship and located in sect. *Verticillata*, suggesting that leaf arrangement appears not suitable as basis for delimitation of subgeneric groups in *Polygonatum*. Additionally, *P. franchetii* is sister to *P. stenophyllum* within sect. *Verticillata*, too. With high morphological and karyological diversity, *Polygonatum* has attracted much attention in phylogenetic and taxonomic research. Our analysis provides more chloroplast genomic information of *Polygonatum* and contributes to improving species identification and phylogenetic studies in further work.

## Supplementary Information

Supplementary Table S1.

Supplementary Table S2.

Supplementary Table S3.

Supplementary Tables.

Supplementary Table S8.

Supplementary Table S9.

Supplementary Table S10.

Supplementary Table S11.

## Data Availability

All data generated or analyzed during this study are included in this published article and the complete chloroplast genome sequences of *Polygonatum campanulatum*, *P. cyrtonema*1, *P. filipes*1, *P. franchetii*, *P. sibiricum*1 and *P. zanlanscianense*1 are deposited in the genbank with ID no: ON534060, ON534061, ON534062, ON534063, ON534064 and ON534059, respectively. Information for other samples used for phylogenetic analysis download from GenBank can be found in Additional Table 1: Table S1.

## Abbreviations

LSC: Large single-copy
SSC: Small single-copy
IR: Inverted repeat
BI: Bayesian inference
ML: Maximum Likelihood
PCGs: Protein-coding genes
CDS: Coding sequence
IGS: Intergenic spacer
rRNA: Ribosomal RNA
tRNA: Transfer RNA
GC: Guanine–cytosine
SSR: Simple sequence repeats
cp: Chloroplast
CTAB: Cetyltrimethylammonium bromide
RSCU: Relative synonymous codon usage
NGS: Next-generation sequencing
