# Supplementary material for: Comparative and phylogenetic analysis of the complete chloroplast genomes of six Polygonatum species (Asparagaceae)
Source: Sci Rep. 2023 May 4;13:7237. doi: 10.1038/s41598-023-34083-1 (PMC10160070; doi:10.1038/s41598-023-34083-1)
Supplement: Supplementary file 4 — Supplementary Tables. [file 41598_2023_34083_MOESM4_ESM.docx]

| **species** | **Repeats type** | | | |
| --- | --- | --- | --- | --- |
|  | **Forward** | **Reverse** | **Complement** | **Palindromic** |
| *Polygonatum campanulatum* | 16 | 2 | 1 | 18  20  17  26  25  22  17  23  23 |
| *Polygonatum franchetii* | 19 | 0 | 0 |  |
| *Polygonatum filipes* | 18 | 1 | 0 |  |
| *Polygonatum zanlanscianense* | 22 | 1 | 0 |  |
| *Polygonatum sibiricum* | 22 | 1 | 0 |  |
| *Polygonatum cyrtonema* | 21 | 1 | 0 |  |
| *Polygonatum kingianum* | 17 | 1 | 0 |  |
| *Heteropolygonatum alternicirrhosum* | 22 | 2 | 0 |  |
| *Heteropolygonatum ginfushanicum* | 20 | 0 | 0 |  |

**Table S4. Repeats types detected in the nine cp genomes**

| **species** | **Size of repeats** | | | | | | |
| --- | --- | --- | --- | --- | --- | --- | --- |
|  | 30–34 | 35–39 | 40–44 | 45–49 | 50–54 | 55–59 | >60 |
| *Polygonatum campanulatum* | 26 | 3 | 1 | 4 | 1 | 0 | 2 |
| *Polygonatum franchetii* | 30 | 2 | 0 | 4 | 1 | 0 | 2 |
| *Polygonatum filipes* | 25 | 3 | 5 | 0 | 1 | 0 | 6 |
| *Polygonatum zanlanscianense* | 26 | 3 | 1 | 5 | 8 | 0 | 6 |
| *Polygonatum sibiricum* | 33 | 3 | 1 | 4 | 1 | 0 | 6 |
| *Polygonatum cyrtonema* | 33 | 3 | 1 | 0 | 1 | 0 | 6 |
| *Polygonatum kingianum* | 25 | 2 | 1 | 0 | 5 | 0 | 2 |
| *Heteropolygonatum alternicirrhosum* | 33 | 2 | 5 | 4 | 1 | 0 | 2 |
| *Heteropolygonatum ginfushanicum* | 29 | 3 | 5 | 4 | 1 | 0 | 1 |

**Table S5. Length of repeats detected in the nine cp genome**

**Table S6. Distribution of repeats detected in functional regions in the nine cp genomes**

| **species** | **Repeats distribution in functional regions** | | | | | | |  |
| --- | --- | --- | --- | --- | --- | --- | --- | --- |
|  | CDS | IGS | Introns | tRNA | IGS/CDS | IGS/  tRNA | IGS/  intron | Intron/  CDS |
| *Polygonatum campanulatum* | 13 | 8 | 5 | 1 | 4 | 3 | 2 | 1 |
| *Polygonatum franchetii* | 18 | 6 | 5 | 1 | 4 | 3 | 2 | 0 |
| *Polygonatum filipes* | 13 | 7 | 6 | 1 | 4 | 3 | 2 | 0 |
| *Polygonatum zanlanscianense* | 35 | 5 | 4 | 1 | 0 | 3 | 1 | 0 |
| *Polygonatum sibiricum* | 28 | 6 | 5 | 1 | 3 | 3 | 2 | 0 |
| *Polygonatum cyrtonema* | 25 | 5 | 4 | 1 | 4 | 3 | 2 | 0 |
| *Polygonatum kingianum* | 16 | 7 | 5 | 1 | 2 | 2 | 2 | 0 |
| *Heteropolygonatum alternicirrhosum* | 24 | 6 | 7 | 1 | 4 | 3 | 2 | 0 |
| *Heteropolygonatum ginfushanicum* | 24 | 4 | 5 | 1 | 4 | 3 | 2 | 0 |

**Table S7. Distribution of repeats detected in regions of the nine cp genomes**

| **species** | **Size of repeats** | | | | |
| --- | --- | --- | --- | --- | --- |
|  | LSC | SSC | IR | LSC/IR | LSC/SSC |
| *Polygonatum campanulatum* | 16 | 3 | 10 | 6 | 2 |
| *Polygonatum franchetii* | 13 | 1 | 19 | 6 | 0 |
| *Polygonatum filipes* | 15 | 3 | 12 | 6 | 0 |
| *Polygonatum zanlanscianense* | 12 | 3 | 30 | 4 | 0 |
| *Polygonatum sibiricum* | 12 | 3 | 27 | 6 | 0 |
| *Polygonatum cyrtonema* | 11 | 3 | 24 | 6 | 0 |
| *Polygonatum kingianum* | 10 | 2 | 16 | 6 | 1 |
| *Heteropolygonatum alternicirrhosum* | 15 | 3 | 23 | 6 | 0 |
| *Heteropolygonatum ginfushanicum* | 11 | 3 | 23 | 6 | 0 |
