# Supplementary material for: Comparative and phylogenetic analysis of the complete chloroplast genomes of six Polygonatum species (Asparagaceae)
Source: Sci Rep. 2023 May 4;13:7237. doi: 10.1038/s41598-023-34083-1 (PMC10160070; doi:10.1038/s41598-023-34083-1)
Supplement: Supplementary file 5 — Supplementary Table S8. [file 41598_2023_34083_MOESM5_ESM.docx]

| **species** | **SSR type** | | | | | |
| --- | --- | --- | --- | --- | --- | --- |
|  | P1 | P2 | P3 | P4 | P5 | P6 |
| *Polygonatum campanulatum* | 38 | 11 | 3 | 7 | 3 | 0 |
| *Polygonatum franchetii* | 37 | 12 | 3 | 6 | 2 | 0 |
| *Polygonatum filipes* | 29 | 10 | 2 | 7 | 2 | 1 |
| *Polygonatum zanlanscianense* | 34 | 9 | 3 | 7 | 2 | 0 |
| *Polygonatum sibiricum* | 30 | 9 | 2 | 7 | 2 | 0 |
| *Polygonatum cyrtonema* | 29 | 9 | 4 | 6 | 2 | 1 |
| *Polygonatum kingianum* | 33 | 11 | 5 | 8 | 2 | 0 |
| *Heteropolygonatum alternicirrhosum* | 39 | 10 | 3 | 8 | 3 | 0 |
| *Heteropolygonatum ginfushanicum* | 34 | 10 | 2 | 7 | 2 | 0 |

**Table S8. The type of SSRs in the cp genome of** **seven *Polygonatum* and two *Heteropolygonatum***
