# Supplementary material for: Comparative and phylogenetic analysis of the complete chloroplast genomes of six Polygonatum species (Asparagaceae)
Source: Sci Rep. 2023 May 4;13:7237. doi: 10.1038/s41598-023-34083-1 (PMC10160070; doi:10.1038/s41598-023-34083-1)
Supplement: Supplementary file 6 — Supplementary Table S9. [file 41598_2023_34083_MOESM6_ESM.docx]

**Table S9.** **The units of SSRs in the nine cp genome**

| SSR | **species** | | | | | | | | | |
| --- | --- | --- | --- | --- | --- | --- | --- | --- | --- | --- |
|  | *P. campanulatum* | *P. franchetii* | | *P. filipes* | *P. zanlanscianense* | *P. sibiricum* | *P. cyrtonema* | *P.*  *kingianum* | *H. alternicirrhosum* | *H. ginfushanicum* |
| A/T | 38 | | 37 | 28 | 32 | 30 | 28 | 33 | 39 | 34 |
| C/G | 0 | | 0 | 1 | 2 | 0 | 1 | 0 | 0 | 0 |
| TA/TA | 5 | | 5 | 4 | 3 | 4 | 4 | 5 | 4 | 4 |
| AT/AT | 3 | | 4 | 3 | 3 | 2 | 3 | 3 | 3 | 3 |
| TC/GA | 3 | | 3 | 3 | 3 | 3 | 2 | 3 | 3 | 3 |
| CAG/CTG | 1 | | 1 | 1 | 1 | 1 | 1 | 1 | 1 | 1 |
| TAA/TTA | 2 | | 2 | 1 | 2 | 1 | 1 | 3 | 1 | 1 |
| AAT/ATT | 0 | | 0 | 0 | 0 | 0 | 1 | 1 | 1 | 0 |
| TAT/ATA | 0 | | 0 | 0 | 0 | 0 | 1 | 0 | 0 | 0 |
| CATT/  AATG | 2 | | 2 | 2 | 2 | 2 | 2 | 2 | 2 | 2 |
| CAAT/  ATTG | 1 | | 0 | 1 | 1 | 0 | 1 | 2 | 1 | 1 |
| AATA/  TATT | 2 | | 2 | 2 | 2 | 2 | 1 | 2 | 1 | 1 |
| GAAT  /ATTC | 1 | | 1 | 1 | 1 | 1 | 1 | 1 | 1 | 1 |
| TTGA  /TCAA | 1 | | 1 | 1 | 1 | 1 | 1 | 1 | 1 | 1 |

| SSR | **species** | | | | | | | | |
| --- | --- | --- | --- | --- | --- | --- | --- | --- | --- |
|  | *P. campanulatum* | *P. franchetii* | *P. filipes* | *P. zanlanscianense* | *P. sibiricum* | *P. cyrtonema* | *P.*  *kingianum* | *H. alternicirrhosum* | *H. ginfushanicum* |
| TTAA/  TTAA | 0 | 0 | 0 | 0 | 0 | 0 | 0 | 1 | 0 |
| TAAA/  TTTA | 0 | 0 | 0 | 0 | 0 | 0 | 0 | 1 | 1 |
| AAAT/  ATTT | 0 | 0 | 0 | 0 | 1 | 0 | 0 | 0 | 0 |
| CGAAA/TTTCG | 2 | 2 | 2 | 2 | 2 | 2 | 2 | 2 | 2 |
| TAATA/  TATTA | 1 | 0 | 0 | 0 | 0 | 0 | 0 | 0 | 0 |
| AATAA/TTATT | 0 | 0 | 0 | 0 | 0 | 0 | 0 | 1 | 0 |
| CAATA/  TATTG | 0 | 0 | 0 | 0 | 0 | 0 | 0 | 1 | 0 |
| GAATAT/ATATTC | 0 | 0 | 1 | 0 | 0 | 0 | 0 | 0 | 0 |
| ATAGTA/TACTAT | 0 | 0 | 0 | 0 | 0 | 1 | 0 | 0 | 0 |

Continuous the table
