# Supplementary material for: Comparative and phylogenetic analysis of the complete chloroplast genomes of six Polygonatum species (Asparagaceae)
Source: Sci Rep. 2023 May 4;13:7237. doi: 10.1038/s41598-023-34083-1 (PMC10160070; doi:10.1038/s41598-023-34083-1)
Supplement: Supplementary file 7 — Supplementary Table S10. [file 41598_2023_34083_MOESM7_ESM.docx]

**Table S10.** **The region of SSRs in the nine cp genome**

| **species** | **Region of microsatelites** | | |
| --- | --- | --- | --- |
|  | **LSC** | **SSC** | **IR** |
| *Polygonatum campanulatum* | 47 | 8 | 8 |
| *Polygonatum franchetii* | 47 | 6 | 7 |
| *Polygonatum filipes* | 38 | 6 | 7 |
| *Polygonatum zanlanscianense* | 41 | 6 | 8 |
| *Polygonatum sibiricum* | 37 | 6 | 7 |
| *Polygonatum cyrtonema* | 34 | 8 | 9 |
| *Polygonatum kingianum* | 42 | 8 | 9 |
| *Heteropolygonatum alternicirrhosum* | 42 | 6 | 14 |
| *Heteropolygonatum ginfushanicum* | 39 | 6 | 11 |
