# Supplementary material for: Comparative and phylogenetic analysis of the complete chloroplast genomes of six Polygonatum species (Asparagaceae)
Source: Sci Rep. 2023 May 4;13:7237. doi: 10.1038/s41598-023-34083-1 (PMC10160070; doi:10.1038/s41598-023-34083-1)
Supplement: Supplementary file 8 — Supplementary Table S11. [file 41598_2023_34083_MOESM8_ESM.docx]

| **M8** | **Gene name** | **Region** | **Selected Sites** | **Pr (w>1)** | **Number of Sites** |
| --- | --- | --- | --- | --- | --- |
| BEB | *psbA* | LSC | 445 N, 556 N, 707 N, 861 F | 0.983*, 0.985*, 0.983*, 0.986* | 4 |
|  | *psbK* | LSC | 3095 W | 0.988* | 1 |
|  | *atpA* | LSC | 4289 Q | 0.984* | 1 |
|  | *rpoC2* | LSC | 9581 A, 9639 V, 9778 I, 9799 M, 9803 I, 9991 E, 9997 A, 10127 Q, 10143 N, 10503 K, 10504 M, 10505 I, 10639 K | 0.984*, 0.984*, 1.000**, 0.986*, 0.985*, 0.987*, 0.987*, 0.987*, 0.983*, 0.985*, 1.000**, 0.984*, 0.984* | 13 |
|  | *rpoC1* | LSC | 11841 R, 12162 E | 0.985*, 0.985* | 2 |
|  | *rpoB* | LSC | 13888 M, 13889 K, 13890 E, 13891 L, 13893 N, 13894 V, 14259 T, 14268 I, 14323 S, 15159 K, 15283 G | 0.988*, 0.983*, 1.000**, 0.987*, 1.000**, 1.000**, 0.985*, 0.983*, 1.000**, 0.984*, 0.987* | 11 |
|  | *psbD* | LSC | 16748 K, 16857 Q | 0.984*, 0.988* | 2 |
|  | *psbC* | LSC | 18626 F | 0.983* | 1 |
|  | *psbZ* | LSC | 19201 L, 19227 F | 0.988*, 0.984* | 2 |
|  | *psaB* | LSC | 20234 A, 21317 Y, 21363 K, 21496 F, 21573 Q, 21577 L, 21818 K | 0.987*, 0.985*, 0.984*, 0.984*, 0.986*, 0.987*, 0.984* | 7 |
|  | *psaA* |  | 21903 R, 21936 I, 21940 I, 22436 P, 22439 Q, 22441 N, 22442 L, 22444 F, 22516 Q, 22707 Y, 23578 K, 23702 G | 0.999**, 0.985*, 0.984*, 0.986*, 0.986*, 0.999**, 1.000**, 0.985*, 1.000**, 0.984*, 0.984*, 0.987* | 12 |
|  | *rps4* |  | 25167 K | 0.984* | 1 |
|  | *ndhJ* |  | 25276 Q | 0.988* | 1 |
|  | *ndhK* |  | 26215 F, 26219 L , 26220 T | 0.999**, 0.964*, 0.996** | 3 |

**Table S11. Positive selected sites detected in the cp genomes of seven *Polygonatum* and two *Heteropolygonatum.***

*: P>95%; **: P>99%)
